# Supramolecular Interactions in Hybrid Polylactide Blends—The Structures, Mechanisms and Properties

**DOI:** 10.3390/molecules25153351

**Published:** 2020-07-23

**Authors:** Anna Kowalewska, Maria Nowacka

**Affiliations:** Centre of Molecular and Macromolecular Studies, Polish Academy of Sciences, Sienkiewicza 112, 90-363 Łódź, Poland; mnowacka@cbmm.lodz.pl

**Keywords:** polylactide, composites, supramolecular interactions, crystallization

## Abstract

The conformation of polylactide (PLA) chains can be adjusted by supramolecular interactions (the formation of hydrogen bonds or host-guest complexes) with appropriate organic molecules. The structures formed due to those intermolecular interactions may act as crystal nuclei in the PLA matrix (“soft templating”). In this review, the properties of several supramolecular nucleating systems based on synthetic organic nucleators (arylamides, hydrazides, and 1,3:2,4-dibenzylidene-d-sorbitol) are compared to those achieved with biobased nucleating agents (orotic acid, humic acids, fulvic acids, nanocellulose, and cyclodextrins) that can also improve the mechanical properties of PLA. The PLA nanocomposites containing both types of nucleating agents/additives are discussed and evaluated in the context of their biomedical applicability.

## 1. Introduction

Polylactide (PLA) is a biodegradable semi-crystalline polymer that has attracted enormous attention over recent years as a biocompatible and environment-friendly material [1,2,3]. It has been approved by the U.S. Food and Drug Administration for biomedical applications and contact with body fluids, e.g., as bioresorbable artificial ligaments or drug delivery systems [4]. PLA is also one of the most important thermoplastic materials for 3D printing [5]. It is easily processable, although its brittleness prevents tensile drawing. The tensile modulus and strength of neat PLA can be increased and the strain at break can be reduced when the polylactide matrix contains a significant amount of crystalline fraction [6]. The crystallinity degree also governs the barrier properties of polylactide [6]. The crystallization behaviour of polylactide has been extensively investigated (details can be found in comprehensive reviews, e.g., [6,7,8]). In general, the type of crystal structure depends on the crystallization conditions. The most common α’- and α-crystals that have similar chain conformations and belong to the same crystal system are formed in melt crystallizations. The less ordered α’-form is obtained exclusively, or as an admixture coexisting with the α-form, when PLA is crystallized isothermally at temperatures below 120 °C. A less thermally stable β-form of a frustrated structure is obtained by stretching the α-form at high draw ratios in the hot-drawing of melt- or solution-spun fibres. The γ-form was obtained by the epitaxial crystallization of PLA on hexamethylbenzene.

Considerable attention was given to the improvement of crystallization kinetics (nucleation and crystal growth) that can be enhanced by nucleators and/or plasticizers. Numerous potential nucleating systems have been examined in the literature, including “green” nucleating agents. PLA-based polymer composites and nanocomposites containing nanoclays [9,10,11], nanosilicas [12,13], carbon nanotubes [14,15], or graphene [16] are well known. Another class of additives for polylactide that can improve its crystallization rate are species that exploit supramolecular phenomena, e.g., hydrogen bonding or host-guest effects, to interact with PLA macromolecules (“soft templating”). Hydrogen bonding is also important for the formation of poly(l-lactide)/poly(d-lactide) (PLLA/PDLA) stereocomplex structures (SC) [17,18,19,20]. The specific C-H···O=C interactions between the paired stereoisomeric PLLA and PDLA chains play a very important role in the formation of SC crystals. It was found that the racemic (3_2_/3_1_) helical conformation of the pair of macromolecules starts to emerge in the melt of a racemic blend, and the formed structures subsequently act as nucleating sites upon cooling [21]. Thus, the conformation of the polylactide chains may be changed by intermolecular interactions with neighbouring macromolecules. It means that the crystallization of the PLA matrix can also be adjusted by hydrogen bonding species, even before the true crystal nuclei emerge. Hydrogen bonding between polylactide and nucleating agents has been postulated, e.g., for PLA blends with amino acids or poly(amino acids) [22], carbon nanotubes [23], phtalimide [24], bisurea derivatives [25], or d-gluconic acid derivatives [26]. PLA was also modified with macromolecular nucleators, such as linear polysilsesquioxanes [27,28,29] or their cyclosiloxane analogues [30] with side substituents acting as donors/acceptors of hydrogen bonds (-OH···O=C-; -COOH···O=C-; and -C-F···H_3_C-) to/from the polyester backbone.

Extremely efficient nucleating systems are based on arylamides and arylhydrazides that may self-organize in the polymer melt. Unfortunately, not all supramolecular nucleators are biodegradable, bioabsorbable, or nontoxic. The potential problems with some of those organic compounds can be a significant concern in the biomedical field. Therefore, polylactide matrices have been also blended with harmless biobased nucleating agents (e.g., orotic acid, humic acids, fulvic acids, nanocellulose, and cyclodextrins). In this review, the research progress on PLA nanocomposites containing both groups of nucleating agents has been evaluated in the context of their biomedical applicability.

## 2. Organic Nucleators

Several groups of organic compounds, such as aryl amide and hydrazide derivatives and relative compounds, can dissolve into PLA melt and then separate and self-organize into supramolecular frameworks upon cooling [31,32,33,34]. The molecular mechanism of such interactions involves hydrogen bonding between C=O in the polylactide macromolecules and the hydrogen bond donating groups (-OH, -NH, and -NH_2_) in the nucleating agents. Those compounds should also have relatively high thermal decomposition temperatures. Self-assembly nucleators have been increasingly used to guide the crystallization of PLA. The heterogeneous nucleation of the polymer matrix is based on crystallization of those organic compounds soluble in PLA melt. The process of organic crystal formation involves nucleation and growth. It makes the crystallite morphology highly dependent on the applied cooling rates, concentrations, and supersaturation. The nucleating efficiency of self-assembling nucleators is largely determined by their surface area-to-volume ratio after crystallization. In the following sections, the PLA nucleation ability of arylamides and hydrazides, orotic acid, and 1,3:2,4-dibenzylidene-d-sorbitol is discussed in comparison to humic acids and fulvic acids.

### 2.1. Arylamide Nucleators

**Scheme 1:**
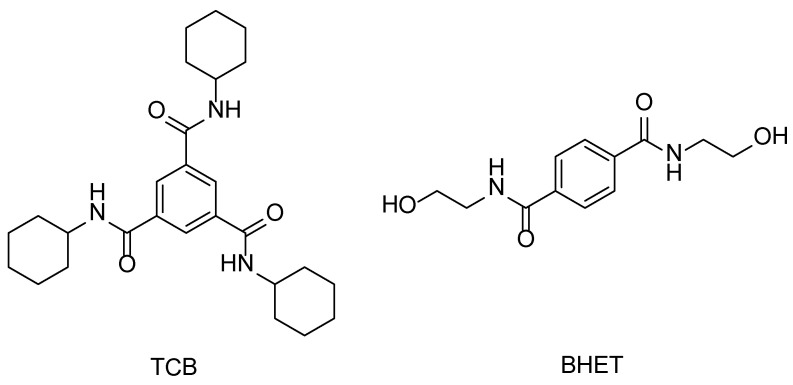
Chemical structures of *N*,*N*′,*N*″-tricyclohexyl-1,3,5-benzenetricarboxylamide (TCB) and *N*,*N*′-bis(2-hydroxyethyl)-terephthalamide (BHET).

The addition of *N*,*N*′,*N*″-tricyclohexyl-1,3,5-benzenetricarboxylamide (TCB, Scheme 1) to PLA can promote the nucleation of the polymer matrix and accelerate the overall crystallization rate [35,36]. The half-time and rate constants of non-isothermal crystallizations carried out at different cooling rates showed that TCB significantly accelerated the process at a very low content of 0.3 wt%. The crystallinity of neat PLA decreased from 47% to 6% when the cooling rate was increased from 1 to 5 °C/min (crystallization did not occur at 10 °C/min), while for PLA nucleated by TCB, it remained almost constant at 45–51% irrespectively of the cooling rates (2.5–10 °C/min).

**Figure 1:**
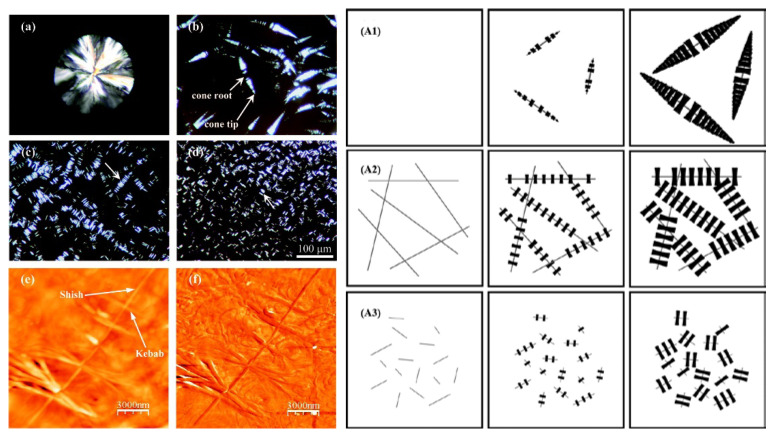
Left panel: Polarized Optical Microscopy (POM) micrographs of the crystal morphology for poly(l-lactide) (PLLA) containing different amounts of *N*,*N*′,*N*″-tricyclohexyl-1,3,5-benzenetricarboxylamide (TCB): (**a**) neat PLLA, (**b**) 0.2 wt%, (**c**) 0.3 wt% and (**d**) 0.5 wt% prepared by isothermal crystallization at 130 °C for 55, 7, 10 and 10 min, respectively, and Atomic Force Microscope (AFM) height (**e**) and phase (**f**) images of PLLA containing 0.2 wt% TCB of a typical shish-kebab-like superstructure. Right panel: schematic representation of the evolution of crystal morphologies during the crystallization of PLLA containing TCB: (**A1**) 0.2 wt%, (**A2**) 0.3 wt% and (**A3**) 0.5 wt% [37]. Reprinted with permission from Macromolecules. Copyright (2011), American Chemical Society.

Three unique crystal superstructures, including cone-like, shish-kebab, and needle-like structures, were obtained by the melt crystallization of PLLA nucleated by TCB [37]. *N*,*N*′,*N*″-tricyclohexyl-1,3,5-benzenetricarboxylamide dissolved in the polymer melt self-organizes upon cooling into fine fibrils prior to PLLA crystallization. The fibrils serve, subsequently, as a “shish” to induce the epitaxial growth (“soft templating”) of “kebab-like” structures approximately orthogonal to the long axis (Figure 1).

**Figure 2:**
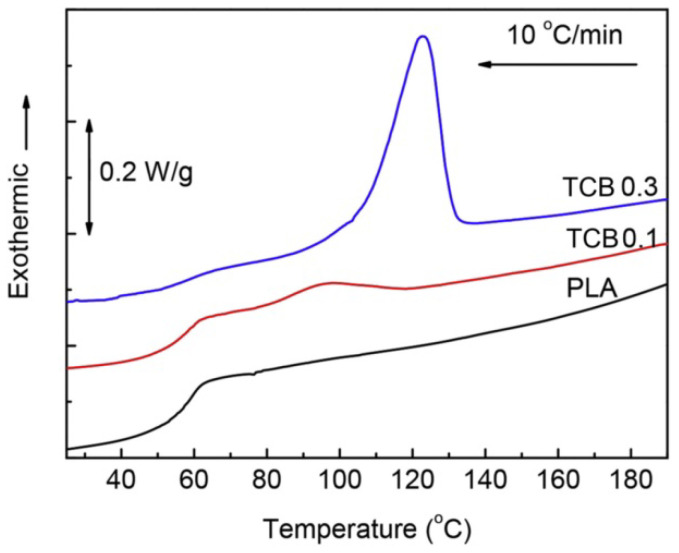
Differential scanning calorimetry (DSC) cooling traces recorded for polylactide (PLA) and its compositions with TCB (0.1 and 0.3 wt%) [38]. Adapted from Polymer Testing. Copyright (2017), with permission from Elsevier.

The effect of TCB on the crystallization behaviour of PLA was studied on a molecular level by time-resolved FTIR and wide angle X-ray diffraction [38]. The observed vibrational changes indicate that the arylamide molecules can accelerate the formation of a skeletal conformational-ordered structure but their presence is more favourable to the formation of a 10_3_ helix structure that is characteristic of α and α’ crystals. The value of the Avrami exponent was lower for PLA nucleated by TCB than that of neat PLA, indicating changes in the crystallization mechanism, although they had no impact on the crystal form. It was also shown that 0.3 wt% of TCB effectively increased the crystallization rate and yield upon cooling from melt at 10 °C/min (Figure 2).

**Figure 3:**
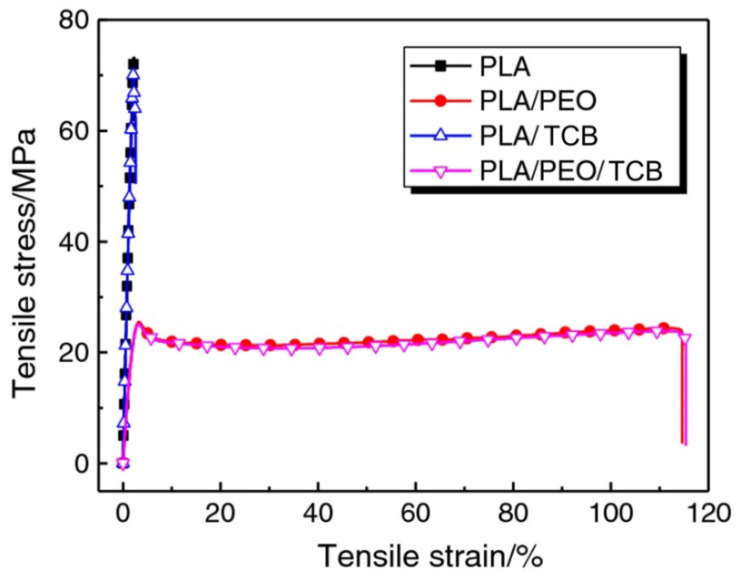
Comparison of tensile stress–strain curves of injection-moulded neat PLA and PLA/poly(ethylene oxide) (PEO) blends with or without TCB [40]. Adapted with permission from Springer Nature: Journal of Thermal Analysis and Calorimetry. Copyright (2018).

The effect of the melting temperatures (ranging from 190 to 240 °C), concentrations of self-assembling TCB (0–0.5 wt%), and cooling rates (2.5–20 °C/min) was investigated for PLA/TCB mixtures [39]. The solubility of TCB was largely dependent on the processing temperature and the concentration of the compound in the polyester matrix. At 240 °C, TCB could dissolve completely and then self-assemble into supramolecular frameworks upon cooling. The crystallization peak temperature of PLA showed a bell-shaped dependence on the concentration of TCB and the cooling rate applied. TCB was also used as a self-assembly nucleating agent for melt-blended PLA/poly(ethylene oxide) (PEO) [40]. Both PEO and TCB exhibited a synergistic effect on promoting PLA crystallization as well as a toughening effect on the blended material (Figure 3). Moreover, TCB prominently reinforced both neat PLA and PLA/PEO blends in the glass transition region and at T > T_g_, indicating an improvement of their heat resistance. The cooperative effect on promoting PLA crystallization was explained by nucleation with TCB and plasticization with PEO chains.

*N*,*N*′-Bis(2-hydroxyethyl)-terephthalamide (BHET, Scheme 1) can be a versatile additive for PLLA, operating both as a plasticizer for processing purposes and as a nucleating agent [41]. BHET crystallizes from the PLA melt during cooling, and the formed crystals facilitate heterogeneous nucleation in the PLA matrix. The formation of BHET crystals with a high surface area-to-volume ratio is favoured at high undercooling/supersaturation. Very importantly, it was proved that the hydroxyl groups of BHET were not involved in transesterification with polylactide chains during extrusion at 200 °C, and the molecular weight of the polyester was not changed. When allowed to crystallize during processing, BHET induced the formation of PLA crystals oriented along the flow direction, enhancing the tensile modulus of the blend. Interestingly, a rapid cooling to T < T_g_ prevented the crystallization of BHET and only a plasticizing effect was indicated by a decrease in both the melt viscosity and glass-transition temperature. A characteristic suppression in the yield point of the amorphous PLA/BHET blend was also observed during mechanical tests with increasing BHET concentration.

### 2.2. Hydrazide Nucleators

**Scheme 2:**
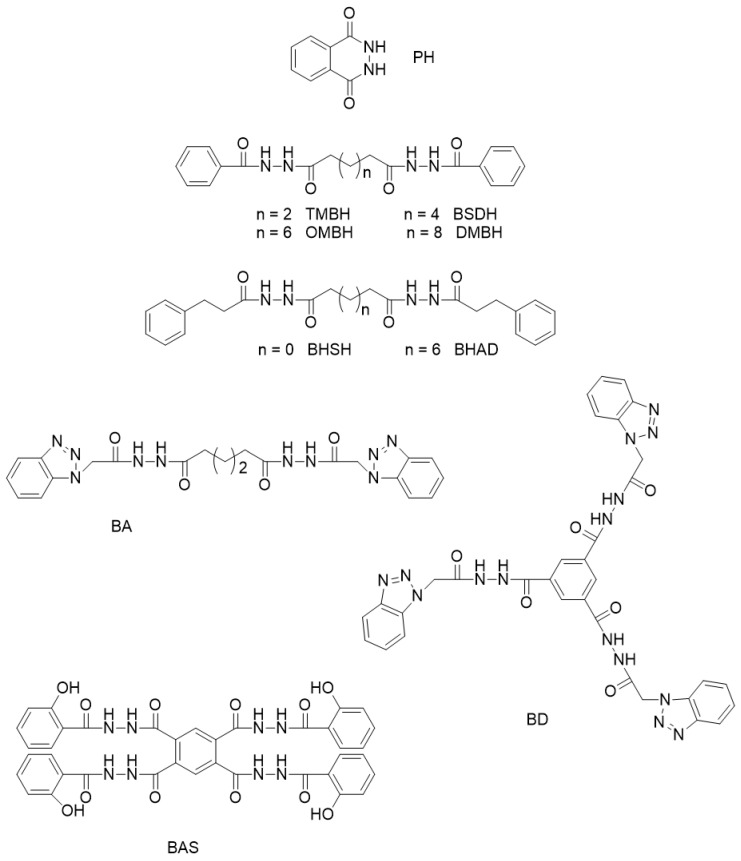
Chemical structures of phthalhydrazide and symmetrical or branched di-, tri-, and tetra-hydrazides.

Aryl hydrazides used as nucleators for the enhancement of polylactide crystallization have two characteristic structural features—a carbohydrazide linkage (-C(O)NH-NHC(O)-) that can take part as an acceptor/donor during the formation of hydrogen bonds, and at least one aryl group providing a system of π-electrons for π-π interactions (Scheme 2). The combination of these two functions allows for an effective supramolecular organization of hydrazide derivatives within polymer melts. Even the small molecule of phthalhydrazide (PH, Scheme 2) can be an efficient nucleating agent that enhances the crystallization of polylactide. It was reported that the non-isothermal melt crystallization of PLA started much earlier in the presence of even a low content of PH (0.1 wt%) [42]. The analysis of Avrami plots indicated that the crystallization mechanism was not changed. The overall effect was ascribed to the increased number of nucleation sites.

However, larger hydrazide derivatives built of at least two aryl groups linked by two carbohydrazide systems separated by an alkyl or aryl spacer are much more effective. They can crystallize within the polymer melt and form structures that can serve as templates for growing polymer crystals. Tetramethylenedicarboxylic dibenzoylhydrazide (TMC, *n* = 2, Scheme 2)—the shortest dihydrazide—exhibited an excellent nucleating effect on PLA. With the addition of 0.05 wt% of TMC, the crystallization half-time of PLA decreased from 26.06 to 6.13 min at 130 °C [43]. TMC did not change the crystallization mechanism in the PLA matrix, as indicated by comparison of the Avrami values. However, the density of nuclei in the PLA was increased, while no discernible effect on the crystalline structure was noted. Non-isothermal crystallization studies indicated that *N*,*N*′-bis(benzoyl)suberic acid dihydrazide (BSDH, *n* = 4, Scheme 2) can accelerate the overall PLLA crystallization rate due to a heterogeneous nucleation effect. With the incorporation of BSDH, the PLLA crystallization peak became sharper and was shifted to a higher temperature range as the sample was cooled down from the melt at a rate of 1 °C/min [44]. The presence of BSDH also affected the isothermal crystalline behaviours (a shorter crystallization time and a faster overall crystallization due to an increased number of small spherulites).

Octamethylenedicarboxylic dibenzoylhydrazide (OMBH, *n* = 6, Scheme 2) was found to be very effective for the acceleration of PLA crystallization under a high cooling rate (50 °C/min with 1 wt% of OMBH) [33]. Very short isothermal crystallization half-times were recorded within 90–130 °C. The moulding cycle time of PLA/OMBH was <3 min. The physical and mechanical properties of the blend were improved as well (a heat distortion temperature of 124 °C, flexural modulus of 4.1 GPa, and Izod impact strength of 7.9 kJ/m^2^). Decamethylenedicarboxylic dibenzoylhydrazide (DMBH, *n* = 8, Scheme 2) also increased the crystallization temperature (T_c_) of PLA [33]. The overall effect of larger dihydrazide molecules is rather complicated and dependent on the crystallization temperature and the crystalline structure. The nucleation efficiency of OMBH significantly depended on its solubility during the thermal annealing (generally increasing with temperature and decreasing with a concentration increase) [45]. However, under certain conditions, the crystallization temperature and nucleation efficiency of OMBH increased with the concentration of the additive, resulting in higher crystallization enthalpy.

**Scheme 3:**
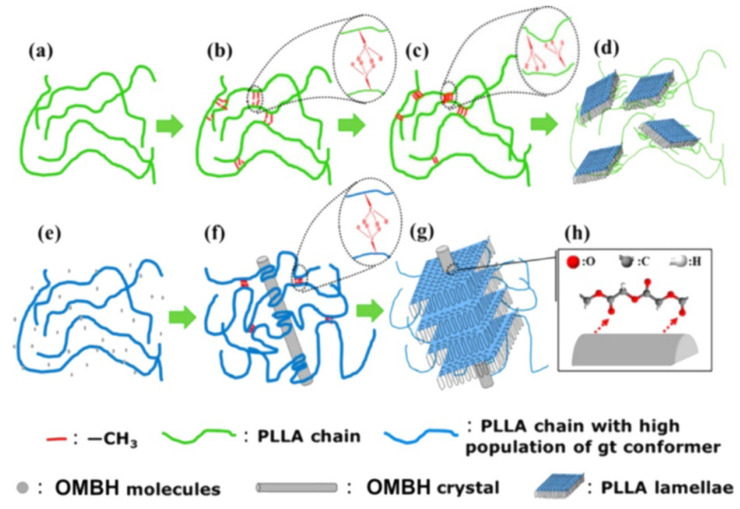
Crystallization of neat PLLA (**a**–**d**) compared to the formation of α-crystals due to “soft templating”, and conformational regulation of PLLA chains through supramolecular interactions with octamethylenedicarboxylic dibenzoylhydrazide (OMBH) (**e**–**h**) [46]. Adapted with permission from Biomacromolecules. Copyright (2017), American Chemical Society.

Time-resolved spectroscopic studies on the interactions between OMBH and polylactide elucidated the nucleation process at the molecular level [46]. The results showed that the crystallization of PLLA in the presence of the dihydrazide nucleator involves not only heterogeneous nucleation with OMBH but also the conformational regulation of polyester chains by hydrogen bonding between the two components of the blend (Scheme 3). The results indicated that due to NH···O=C interactions between the dissolved nucleator and PLLA, the building blocks of the PLLA chains were transformed into trans-gauche conformers before the self-assembling of OMBH into nanocrystals and their phase-separation from the PLLA melt. It resulted in a decrease in the energy barrier to the formation of α-crystals of polylactide. Once the dissolved molecules of OMBH start to self-assemble into nanostructures upon cooling, the polylactide chains with an increased population of trans-gauche conformers begin to form primary nuclei on the surface of OMBH nanofibrils and crystals. Therefore, conformational regulation was proposed for the crystalline manipulation of PLLA by hydrazide nucleators.

**Figure 4:**
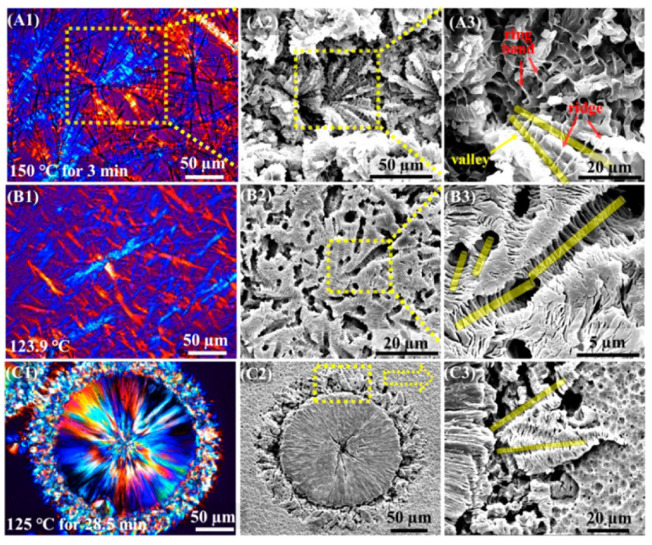
POM (**A**1–**C**1) and SEM micrographs (**A**2–**C**2 and **A3**–**C**3) of the crystalline superstructures formed by PLLA in the presence of OMBH (A: 1 wt%; B: 0.5 wt%; C: 0.3 wt%) after different thermal treatments [47]. Reprinted with permission from Biomacromolecules. Copyright (2017), American Chemical Society.

It was also shown that OMBH can self-assemble into diverse frameworks that induce the formation of various crystalline superstructures of polylactide, depending on their content in the polymer melt and the processing conditions (Figure 4). Larger amounts of OMBH (1 wt%) first self-assembled into star multiarm frameworks (at 170 °C), and then each of the resultant arms served as a “shish” to induce the growth of PLLA lamellae after cooling down to 150 °C. The nucleating sites on the surface of such structures are scarce, and thus, the lamellae grew as branched “calabash” structures. A slightly lower amount of OMBH (0.5 wt%) self-assembled at 125.3 °C into short fibril-like frameworks with a sufficient number of available nucleating sites. It resulted in a transcrystalline superstructure with PLLA crystals growing epitaxially orthogonally to the long axis of the OMBH fibrils. An interesting sunflower-like superstructure with big PLLA spherulite centres, surrounded by hybrid fibril-like trans-crystalline superstructures at the boundary between the spherulite and the amorphous region, was obtained in the presence of a very small amount of OMBH (0.3 wt%).

**Figure 5:**
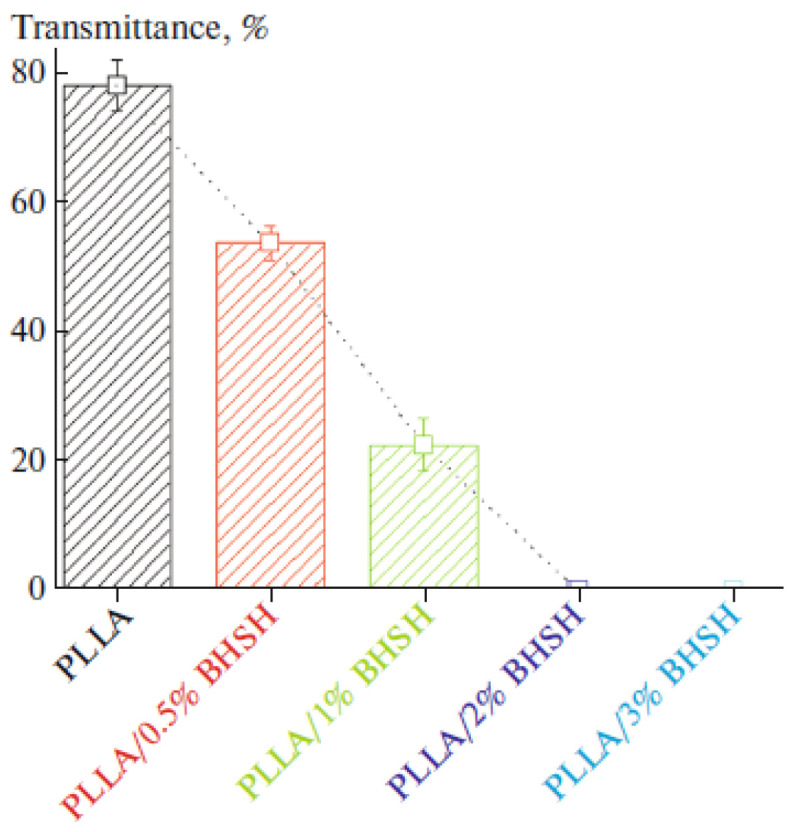
Light transmittance of PLLA and its composites with bis(hydrocinnamic acid) dihydrazide (BHSH) [47]. Reprinted with permission from Springer Nature: Polymer Science, Series A. Copyright (2019).

The crystallization of polylactive was also studied in the presence of more structurally complicated dihydrazides. The studies revealed that both the cooling rate and the melting temperature affected the non-isothermal crystallization behaviour of PLLA in the presence of *N*,*N*′-succinic bis(hydrocinnamic acid) dihydrazide (BHSH, Scheme 2) [47] and *N*,*N*′-sebacic bis(hydrocinnamic acid) dihydrazide (HAD, Scheme 2) [48]. BHSH accelerated both the melt crystallization and the non-isothermal crystallization of PLLA. At a content of 2 wt%, the half time of crystallization at 100 °C decreased by twelve times, compared to that of neat PLLA (48.6 vs. 575.7 s). HAD (1 wt%) increased both the crystallization temperature and non-isothermal crystallization enthalpy from 94.5 °C and 0.1 J/g (neat PLLA) to 131.6 °C and 48.5 J/g, respectively. It was also found that the cold crystallization behaviour of PLLA/HAD was almost independent of the HAD concentration, when it was larger than 2 wt%. Although those compounds vastly improved the crystallization of PLLA, their presence decreased the thermal stability and light transmittance of the PLLA films (Figure 5). This effect was ascribed to the increased crystallinity of the blends as well as the colour of the additives.

The non-isothermal crystallization of PLLA was also significantly promoted by the presence of 1 wt% of *N*,*N*′-bis(1H-benzotriazole) adipic acid acethydrazide (BA, Scheme 2) [49], 0.5 wt% of *N*,*N*,*N*′-tris(1H-benzotriazole) trimesinic acid acethydrazide (BD, Scheme 2) [50], and 3 wt% of *N*,*N*,*N*,*N*′-salicylic tetra(1,2,4,5-benzenetetracarboxylic acid) hydrazide (BAS, Scheme 2) [51]. Upon cooling at a rate of 1 °C/min, the onset of crystallization temperature was shifted from 101.4 to 111.3 °C upon the addition of 0.5 wt% BD to the PLLA. The enthalpy of non-isothermal crystallization increased from 0.1 to 38.6 J/g. The isothermal crystallization half-time decreased as well. An enhanced nucleation density was indicated by double-melting peaks of PLLA/0.5% BD, assigned to melting-recrystallization. The equilibrium melting point of the PLLA/0.5% BD blend was set at lower temperatures than those of neat PLLA. A similar effect was observed for blends of PLLA and BAS. This additive is a highly efficient nucleating agent that can significantly promote the crystallization of PLLA upon cooling at a rate of 20 °C/min. The best acceleration of the melt crystallization of PLLA carried out upon cooling at 1 °C/min was achieved with 3 wt% of BAS. The final melting temperature was another important factor. The best temperature for the melt-crystallization of the blend was 200 °C. Despite the improvement of the thermal stability and fluidity compared with the neat PLLA, a decrease in the light transmittance through the PLLA/BAS blends was observed.

**Scheme 4:**
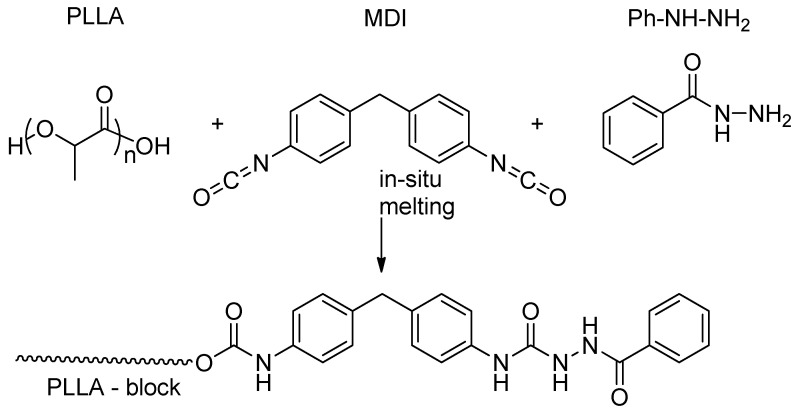
Postulated pathway of the reaction between 4,4′-diphenylmethane diisocyanate, benzohydrazine, and PLLA.

Hydrazide nucleating species were also obtained by an in situ reaction between 4,4′-diphenylmethane diisocyanate (MDI) and benzohydrazine (P) in the polylactide matrix (Scheme 4) [52]. The rate of PLLA crystallization was enhanced by blending those reagents with the polyester upon melting. The reaction between the components was confirmed by NMR spectroscopy. This procedure increased the compatibility between the nucleating agents and the polylactide matrix. The crystallinity of the PLLA increased from 10.3% to 42.1% upon adding 0.25% (MDI + P) and melting them for 8 min. The PLLA crystallization half-time at 130 °C decreased from 42.0 to 1.1 min. Moreover, the heat resistance of the PLA was enhanced and good mechanical properties were achieved. 

### 2.3. PLA/Orotic Acid Blends

**Scheme 5:**
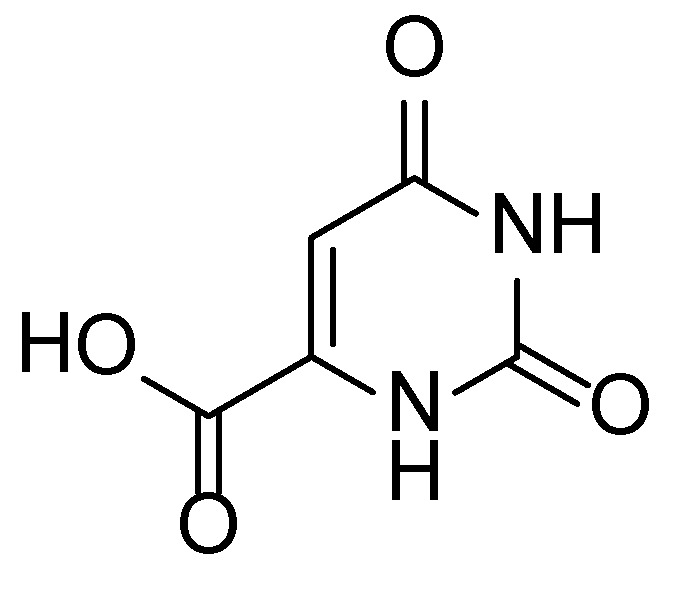
Chemical structure of 2,6-dioxo-1,2,3,6-tetrahydropyrimidine-4-carboxylic acid (orotic acid).

Orotic acid (2,6-dioxo-1,2,3,6-tetrahydropyrimidine-4-carboxylic acid, OA) (Scheme 5) is a compound synthesized by living organisms as a key substance in the biosynthesis of naturally occurring pyrimidines [53,54]. Owing to its hydrogen-bond donating/accepting properties, it may also serve as an excellent biobased nucleating agent for polylactide. The temperature of the melting of OA (345–346 °C) is high enough to allow melt compounding with PLA.

**Figure 6:**
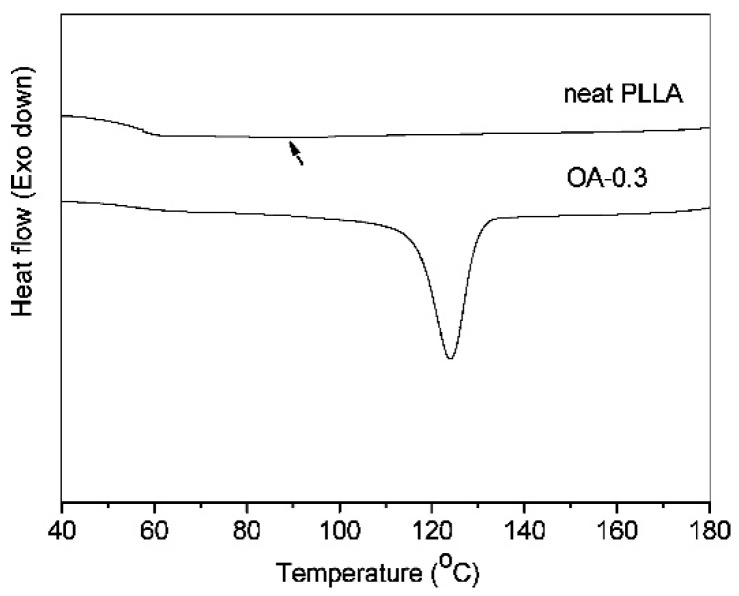
A comparison of DSC cooling (10 °C/min) traces of neat PLLA and the composite containing 0.3 wt% of OA (OA-0.3) [55]. Reprinted with permission from Industrial & Engineering Chemistry Research. Copyright (2011), American Chemical Society.

Both the non-isothermal and isothermal melt crystallization kinetics of PLLA were enhanced significantly by orotic acid, even at a very low content (0.3 wt%) [55]. The presence of OA effectively induced the crystallization of PLLA upon fast cooling (10 °C/min) (Figure 6). The crystallization peak temperature was 123.9 °C and the crystallization enthalpy was equal to 34.1 J/g when the sample was crystallized non-isothermally from melt at a cooling rate of 10 °C/min. The half-time of isothermal crystallization at 120 °C was 0.64 min.

**Figure 7:**
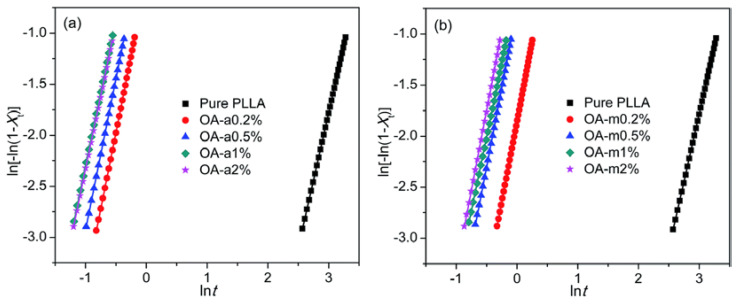
Avrami plots for blends of PLLA and (**a**) anhydrous or (**b**) monohydrate orotic acid isothermally melt-crystallized at 130 °C [56]. Published by The Royal Society of Chemistry (2017).

Tests with anhydrous (OA-a) and monohydrated (OA-m) orotic acid were carried out to gain an insight into the role that water molecules play in the activity of orotic acid as a nucleating agent in PLA crystallization [56]. The compounds were mixed with PLLA melt, and their nucleation effectiveness in the non-isothermal and isothermal melt crystallization of PLLA was investigated. Although OA-a showed more prominent nucleation efficiency than OA-m, both forms of orotic acid improved the nucleation density, the degree of crystallinity of the PLLA, and the overall crystallization rate. The data derived from Avrami plots (Figure 7) suggest a simultaneous nucleation and a three-dimensional crystal growth. The number of spherulite nucleation sites increases with increasing OA content, while their size decreases. The Avrami exponent *n*, indicating the nature of nucleation and the dimensionality of crystal growth, is very close to 3 regardless of the degree of orotic acid hydration. Nevertheless, the molecules of water bound to OA-m and its dehydration transition seem to decrease the nucleation effect. The nucleation density is higher and the spherulite size distribution is more uniform in PLLA/OA-a blends. This can be linked to the better dispersion and more uniform distribution of active nucleating agents in the blends.

The isothermal crystallization behaviour of PLLA/OA blends was investigated with time-resolved FTIR spectroscopy [56]. The spectral evolution of conformational changes and chain packing during thermal annealing at 140 °C showed that for both PLLA admixed with OA-a and OA-m, the crystallization proceeded with changes in the interchain interactions that were followed by the formation of the 10_3_ helix. However, the induction time and crystallization half-times were much shorter for the sample containing anhydrous OA. The formation of crystal structure is similar to that observed for pure PLLA at, for example, 120 °C, despite the fact that without OA, PLLA hardly crystallized at 140 °C. The photodegradation and biodegradation of PLA in the presence of orotic acid were also studied [57]. Rheological measurements showed that OA slightly enhanced the UV-induced cleavage of polyester chains. Photodegradation was shown to accelerate the subsequent biodegradation, as macromolecules of smaller molecular weight are more prone to decomposition. Orotic acid itself promoted biodegradation both in native and photodegraded samples, by shortening the lag phase and increasing the rate of the process.

### 2.4. PLA/Sorbitol Blends

**Scheme 6:**
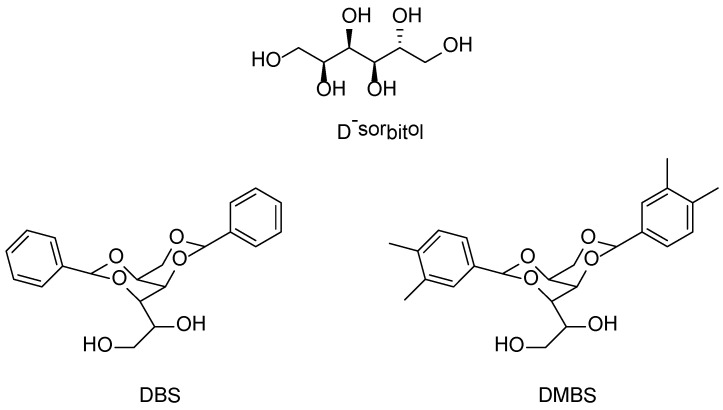
Chemical structure of d-sorbitol, 1,3:2,4-dibenzylidene-d-sorbitol (DBS), and 1,3,2,4-bis(3,4-dimethylobenzylideno)sorbitol (DMBS).

1,3:2,4-Bibenzylidene-d-sorbitol (DBS, Scheme 6), derived from the natural sugar alcohol d-glucitol, is considered an environmentally friendly material of acceptably low toxicity. It is a well-known low-molecular-weight supramolecular gelator of “butterfly-like” structure that has considerable potential in applications such as personal care products, adhesives, dental composites, gel electrolytes, or liquid crystalline materials and as a polymer nucleator [58].

The benzylidene groups and hydroxyl moieties on the sorbitol backbone are two molecular recognition motifs underpinning the role of DBS in the formation of a nanoscale network and the gelation mechanism. It involves interactions between 5-OH/6-OH hydrogen-bond-donating groups of one molecule of DBS and the hydrogen-bond-accepting 5-OH/6-OH or the cyclic acetals of another molecule. The π-π stacking/solvophobic interactions between the aromatic substituents are other important factor in the molecular recognition pathway. In non-polar media, the intermolecular hydrogen bonding plays a key role in the self-assembly, whereas in polar, protic solvents, the π-π stacking and/or solvophobic interactions between the aromatic groups become more important. The primary one-dimensional nanostructures (nanofibrils) formed by DBS molecules bundle together into 3D nanofibers (10 nm to 0.8 μm in diameter) of helical or non-helical structure (depending on the environment’s polarity), and a three-dimensional network is subsequently formed.

**Scheme 7:**
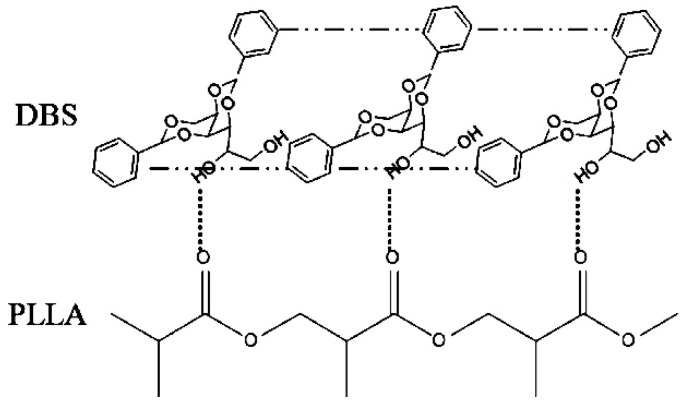
Postulated interactions between the polylactide backbone and nanofibrils of self-assembled DBS [59]. Reprinted with permission from The Journal of Physical Chemistry B. Copyright (2011), American Chemical Society.

DBS can direct the crystallization of polylactide. It influenced the crystal structure of PLLA and affected its crystallization rate and melting behaviour. FTIR spectroscopy showed that the C=O stretching band in the PLLA sample at 1755 cm^−1^ became much broader as DBS was added, presumably due to hydrogen bonding between DBS and PLLA [52]. In the presence of DBS, the formation of the perfect α-form was favoured over the less ordered α’-structures [59]. The α-crystals were obtained more easily, and the disorder-to-order (α’-to-α) phase-transition was shifted to lower temperatures with an increasing concentration of DBS. This was explained in terms of supramolecular templating exerted by DBS nanofibrils. DBS molecules stacked together through π-π interactions to form a strand that was linked to PLLA macromolecules by hydrogen bonding (Scheme 7). The equilibrium melting point and glass-transition temperature of PLLA admixed with DBS were not significantly changed.

**Figure 8:**
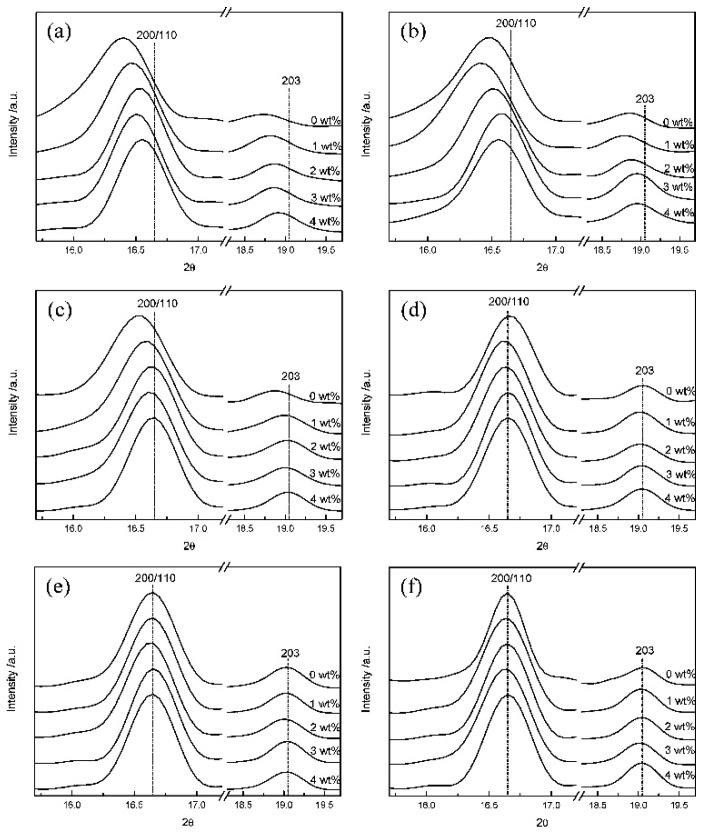
Wide-angle X-ray Scattering (WAXS) patterns of PLLA containing 0–4 wt% of DBS isothermally crystallized at (**a**) 90, (**b**) 100, (**c**) 110, (**d**) 120, (**e**) 130, and (**f**) 140 °C [59]. Reprinted with permission from The Journal of Physical Chemistry B. Copyright (2011), American Chemical Society.

The X-ray diffraction peaks at 2θ = 12.5°, 14.7°, 16.7°, 19.1° and 22.4° correspond to the (103), (010), (200)/(110), (203) and (015) reflections characteristic of the α-crystals of PLLA, whereas the one at 2θ = 24.5° is representative of the less perfect α’-crystals. The latter are typically formed as a single form or coexist with α-crystals at crystallization temperatures <120 °C. It was found that with an increase in DBS content, the reflections (200)/(110) moved forward to the higher 2θ range and, contrary to the behaviour of neat PLLA, they were not significantly changed during isothermal crystallization at 120, 130 and 140 °C (Figure 8) [59]. The α-crystals predominate even at T < 120 °C. The temperature of the α’-to-α phase-transition of PLLA was lowered with an increase in the DBS concentration, owing to the interactions between the polyester chains and the self-organized nanofibrils.

**Figure 9:**
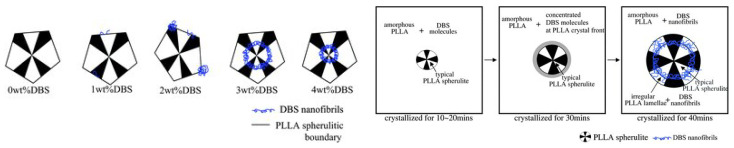
Concentration-dependent formation of nanofibrils of DBS during crystallization of PLLA [52]. Republished with permission of Royal Society of Chemistry. Permission conveyed through Copyright Clearance Center, Inc.

The DBS architectures could be tuned by varying the DBS contents and PLLA crystallization temperatures [52]. Micron-sized fibrillar rings or disks and “concentric-circled” PLLA spherulites were formed due to the aggregation of nanofibrils in samples with DBS contents >3 wt% crystallized above 120 °C (Figure 9). The dispersed nanofibrils affected the orientation of PLLA lamellae and caused a change in birefringence, not significantly affecting the spherulitic growth rate. If the local concentration of DBS was low then PLLA crystallized in a typical manner and DBS molecules were excluded outside the crystals. Their concentration in the amorphous region increased. When the PLLA crystallization was complete, the concentration of the DBS molecules ejected outside the PLLA crystals became high enough to let them self-assemble at the PLLA growth front and form nanofibrils between the spherulites. For higher DBS contents (3–4 wt%) the formation of nanofibrils was much easier. Therefore, they were formed during the process of PLLA crystallization and aggregated inside the spherulites. More regular PLLA crystals were formed at lower temperatures when larger amounts of DBS were added [60]. Moreover, the spherulitic growth rate of PLLA depended inversely on the fold surface free energy, which increased with the amount of the additive. DBS also enhanced the hardness and stiffness of the PLLA on cooling [61]. Furthermore, the hydrophilicity of the PLLA was significantly improved by an increase in the DBS concentration, which is important for biomedical applications of PLA. The crystallization of the PLA melt was also enhanced in a more complex blend, via the synergistic effect of DBS nanofibrils as the nucleating agent, combined with a plasticizer (poly(ethylene glycol), PEG) and a multifunctional monomer (pentaerythritol triacrylate, PETA) [62]. It was crucial to prepare the DBS/PEG gel before mixing those components with PLA. The mixing temperature was also critical. The acceleration of crystallization was ascribed to the increase in the nucleation density as well as the faster growth rate of spherulites in the presence of the plasticizer. 

An analogue of DBS—1,3,2,4-bis(3,4-dimethylobenzylideno)sorbitol (DMBS) (Scheme 6)—was applied to poly(lactic acid) as a nucleating and clarifying agent [63]. The nucleated PLA crystallized earlier, and a reduction in the crystallization temperature was observed at DMBS concentrations of 0.25–10 wt%, while the glass transition temperature was decreased by ~10 °C. The composite maintained similar clarity at all DMBS concentrations. The tensile modulus and tensile strength increased slightly with the content of DMBS up to 1.5 wt% but dropped at higher concentrations of the additive. Biodegradable PLA composites were prepared using a combination of wood fiber (WF) and DMBS [64]. 1,3,2,4-bis(3,4-dimethylobenzylideno)sorbitol acted as an effective nucleating agent for those composites, improved their thermal stability and mechanical properties, and slowed down their enzymatic degradation.

### 2.5. PLA/Humic Substances

**Scheme 8:**
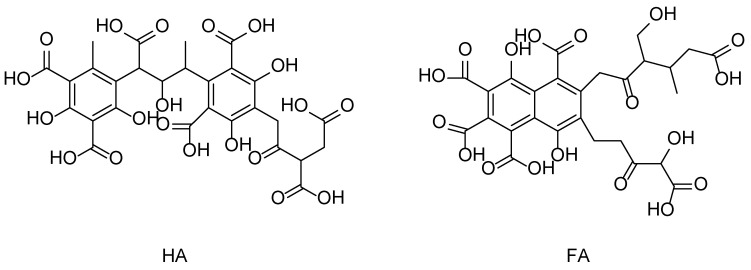
Exemplary components of Humic Acids (HA) and Fulvic Acids (FA).

Humic substances (HS) are organic matter distributed in the natural environment. They comprise a complex mixture of humic acids (HA), fulvic acid (FA), and humins (Scheme 8) [65]. Humic substances can be considered as environmentally and biologically safe natural antioxidants. Blends of HA/FA and PLA are interesting because of their potential applicability as biomedical scaffolds or packaging materials for oxidation-sensitive food. Owing to its biological origin, a black mixture of HA has an undefined composition that may vary depending on their origin and the process of extraction. The components generally contain aromatic and aliphatic structures with attached carbonyl, carboxylic, and phenolic groups, as well as a minor amount of amine and amide residues that may contribute as donors/acceptors for the formation of hydrogen bonds. Thus, HA may display interfacial activity, hydrophilicity, and cation exchange and complexation capacities. HA have an amphiphilic character and are soluble in aqueous alkaline media. They are not soluble in neutral to acidic media and form micelle-like structures under such conditions. Fulvic acids are the yellowish brown, low molecular weight fraction of the amorphous polymeric mixture of humic acids. FA are soluble in aqueous media of different pH as well as in some organic solvents (acetone and ethanol).

The polycondensation of L-lactic acid was performed at 150 °C in the presence of HA (0.01% *w*/*v*), resulting in a 93% yield of a hybrid polymer of molecular weight 6.4 × 10^5^ g/mol [66]. The incorporation of HA slightly enhanced the thermal stability of the polyester matrix. The glass transition temperature and the temperature of melting were reduced, although the elongation at break and ductility were enhanced. The presence of HA in the blend significantly improved the hydrophilicity of the polyester film, total content of phenolics (0.075 µmol GAE/g film), and water absorption capacity (90.65%). An improvement in the antioxidant activities and radical scavenging properties was also observed.

**Figure 10:**
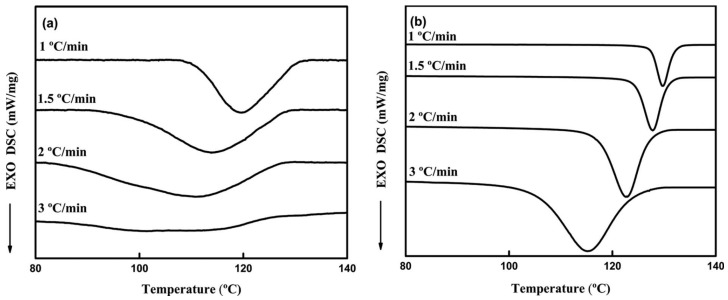
DSC thermograms of non-isothermal crystallization for PLA (**a**) and PLA containing 0.3 wt% of HA-amide-1 (**b**) [68]. Reprinted with permission from Springer Nature. Copyright (2017).

**Figure 11:**
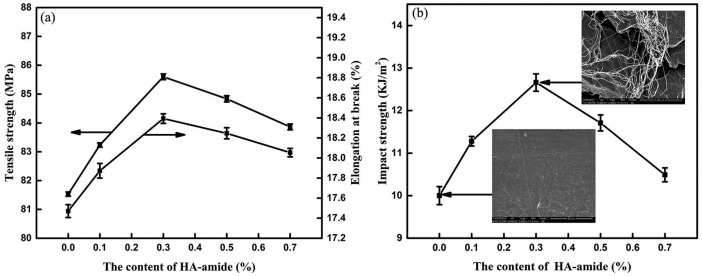
Effects of HA-amide-1 on mechanical performance of PLA blends: (**a**) tensile strength and elongation at break; (**b**) impact strength (insets: impact fracture SEM images) [67]. Reprinted with the permission of the publisher (Taylor & Francis Ltd., http://www.tandfonline.com).

Humic acids may be also derivatized to increase their compatibility with the PLA matrix in the hybrid blends. For example, the amount of amide groups was increased by the amidation of HA using dodecylamine in the presence of carbonyl diimidazole as the coupling agent (HA-amide-1) [67] or aniline in the presence of a phosphorus trichloride catalyst (HA-amide-2) [68]. The non-isothermal crystallization kinetics showed that HA amides incorporated during the thermoplastic processing may serve as nucleating agents that enhance the crystallization rate of PLA. Inclusions of HA amide provided a large number of heterogeneous nucleation sites thus increasing the degree of the crystallinity of the PLA/HA-amide composites (Figure 10). The crystallization behaviour varied under different cooling conditions. The mechanical properties of the blends were also improved after the introduction of HA amides that increased the rigidity of the network structure in the PLA matrix (Figure 11).

Analogously to HA, FA structural units consist of polycyclic aromatic structures and functional groups (e.g., -COOH, -OH, R-CH=CH-OH, and C=O). They can thus be modified through sulfonation, nitration, etc. reactions in order to broaden the application scope. Fulvic acids have also been transformed into amide derivatives in order to augment their interactions with polylactide chains. Fulvic acid amide (FAA) was synthesized with fulvic acids and urea [69]. FA derivatives were also prepared by coupling FA with benzhydrazide (FA-BH) [70] or p-phenylenediamine (MFA) [71]. 

**Figure 12:**
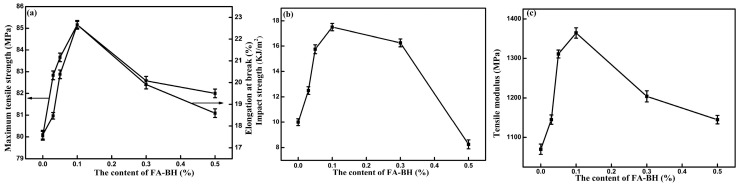
Effects of fulvic acid–benzhydrazide (FA-BH) on mechanical properties of PLA-based composites: (**a**) maximum tensile strength and elongation at break; (**b**) impact strength; (**c**) tensile modulus [70]. Reproduced with permission of John Wiley & Sons Ltd. from Liu P., Zhen W., Bian S., Wang X., Advances in Polymer Technology, 2018; 37, 2788–2798.

**Figure 13:**
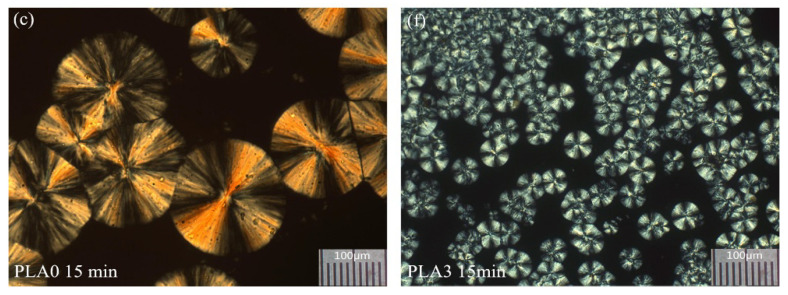
Evolution of spherulitic structures in PLA (**c**) and PLA3 (0.1 wt%) (**f**) melted at 200 °C and then crystallized at 130 °C for 15 min, observed with polarized optical microscopy [70]. Reproduced with permission of John Wiley & Sons Ltd.

**Figure 14:**
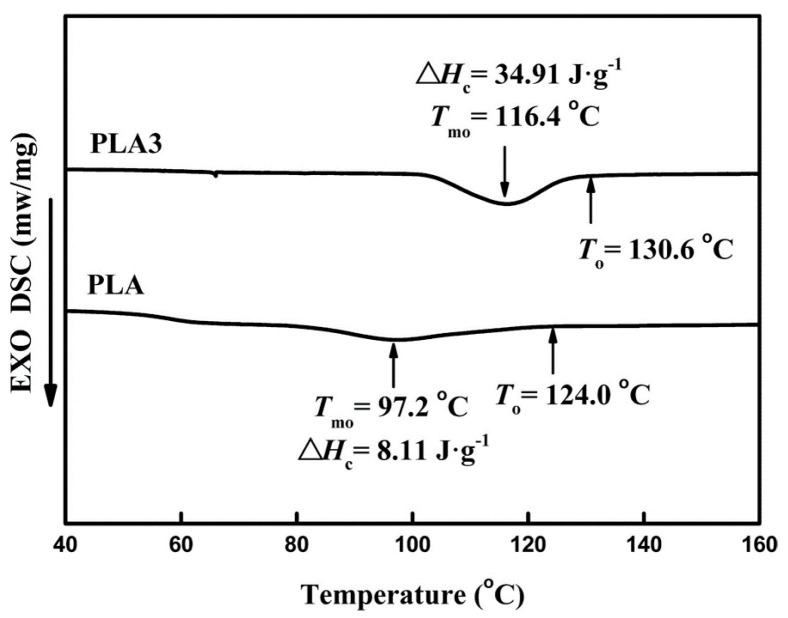
Thermal characteristics of PLA and PLA/FA-BH hybrid blend (PLA3) containing 0.1 wt% of the additive upon cooling at 2 °C/min [70]. Reproduced with permission of John Wiley & Sons Ltd. Copyright (2018).

Polylactide blends containing the derivatives of fulvic acids were obtained upon melt blending. FAA accelerated the PLA crystallization rate and improved toughness of the PLA/FAA composites [69]. FAA acted as a heterogeneous nucleation agent and enhanced the three-dimensional spherulitic crystal growth in PLA. The nucleating mechanism of PLA/FAA proposed to explain the observed phenomena was based on the hydrogen bonding between C=O in the polyester backbone and N-H residues in FA derivatives. The rheological behaviour of the PLA/FAA blends showed an increase in the storage modulus induced by FAA. The apparent viscosities and thermal stability of the composites were much higher after the blending. The rheological behaviour indicated good interfacial compatibility between PLA and FA grafted with *p*-phenylenediamine (MFA) [71]. Mechanical tests showed that the impact strength of PLA admixed with 0.5 wt% MFA was improved by 97.2% and the impact destruction resulted in the characteristic ductile fracture. Moreover, the rate of nucleation was improved and the crystallinity of the PLA increased from 4.9% to 36.9% in the presence of MFA. Interestingly, MFA also influenced the mechanism of enzymatic degradation, increasing the Km of proteinase K and somewhat inhibiting the degradation. The mechanical performance of composites containing 0.1 wt% FA-BH was improved [70]. The network structure of the PLA/FA-BH blend was more rigid. The tensile strength, tensile modulus, elongation at break, and impact strength increased, respectively, by 6.38%, 27.47%, 28.75% and 74.56% (Figure 12). However, larger amounts of FA-BH deteriorated the properties of the PLA. FA-BH acted efficiently as nucleating agents (Figure 13). The crystallization rate of the PLA matrix was improved by 0.1 wt% of FA-BH. The degree of crystallinity increased to 41.88% (Figure 14) and the temperature of crystallization increased from 97.2 to 116.4 °C upon cooling from melt.

**Scheme 9:**
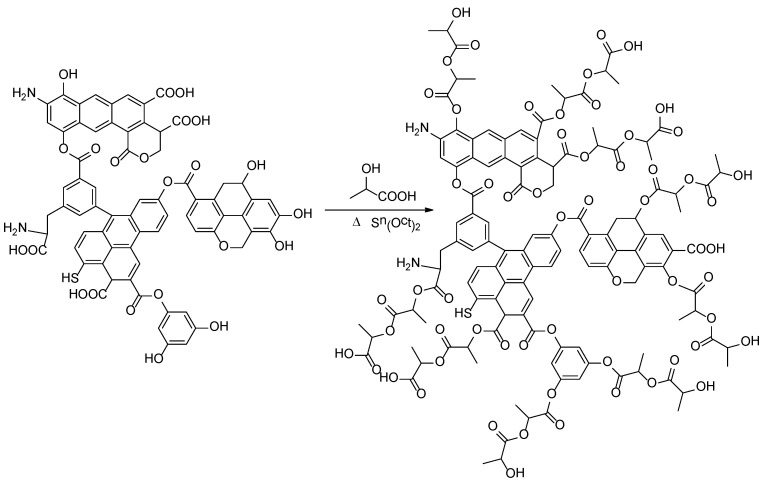
Synthesis of copolymer PLA-FA.

**Figure 15:**
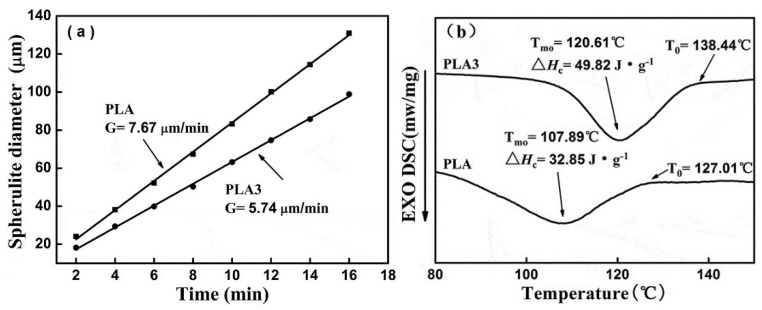
(**a**) The spherulite growth rate of PLA and 0.5 wt% of PLA-FA (PLA3) and (**b**) cooling DSC curve of PLA and PLA3 [72]. Reprinted by permission of the publisher Taylor & Francis Ltd. Copyright (2019).

Polylactide has been also modified with an FA-based hybrid macromolecular nucleator—a poly(lactic acid)-fulvic acid graft copolymer (PLA-FA) [72]. PLA-FA was synthesized with lactic acid monomer and FA as shown in Scheme 9. The obtained hybrid polymers had molecular weight (Mw) of about 14,300 g/mol and polydispersity index (PDI) = 1.3. The narrow molecular weight distribution implies that the grafted PLA chains were rather short and the PLA-FA macromolecules had a compact quasi-spherical structure. The presence of polyester components improved the compatibility of FA with the PLA matrix. The structure characterization and tests demonstrated that the PLA-FA used as a hybrid filler effectively enhanced the performance of the PLA composites prepared by melt blending. A plasticization effect of PLA-FA was indicated by the results of rheological analysis. Various plasticizers of low or high molecular weight are well known for the improvement of ductility, flexibility, and processability of PLA due to the increased number of independently moving segments [6]. Plasticisation can also be beneficial for the growth of crystals in amorphous matrices. Consequently, the PLA-FA additive promoted not only the nucleation (Figure 15) but also the rate of the non-isothermal crystallization of the PLA composites and improved their thermal stability and toughness.

**Figure 16:**
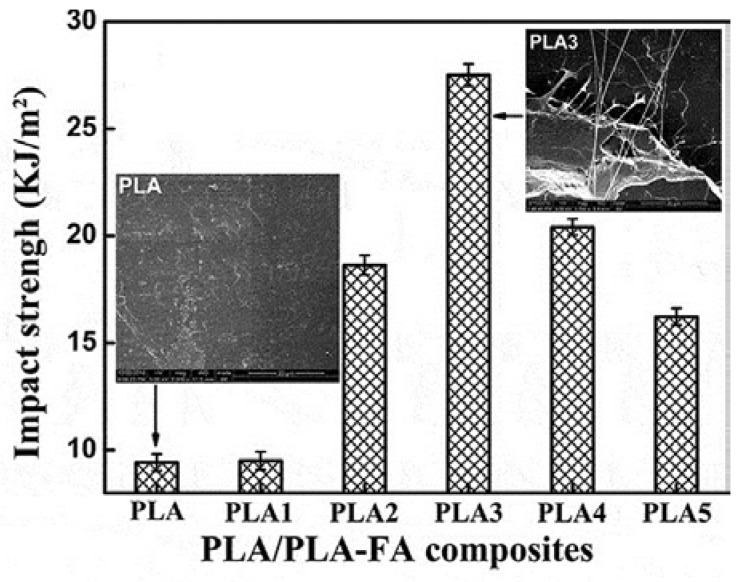
The impact strength (inset: impact fracture SEM images) of PLA/PLA-FA composites (PLA1: 0.1 wt%; PLA2: 0.3 wt%; PLA3: 0.5 wt%; PLA4: 0.7 wt%; PLA5: 1 wt% of PLA-FA) [72]. Reprinted by permission of the publisher Taylor & Francis Ltd. (http://www.tandfonline.com).

The effect of PLA-FA on the properties of polylactide blends can be related to the amount of the macromolecular additive. It affected not only the crystallization process but also the thermal and mechanical properties of the composite. The toughness of the PLA matrix was improved, while its strength and rigidity were enhanced. The sample containing 0.5 wt% of PLA-FA (PLA3) had the best properties (Figure 16). A significant increase in ductility and flexibility was noted for PLA3. The impact strength of this sample was improved by almost 200% compared with pure PLA. This is larger than the increase in impact strength induced by amidated FA (FAA, FA-BH, or MFA). However, when the amount of PLA-FA exceeded 0.5%, the mechanical properties of the blends gradually decreased. The effect was attributed to the agglomeration of the additive in the polylactide matrix, which led to stress concentration.

## 3. PLA/Nanocellulose Composites

**Figure 17:**
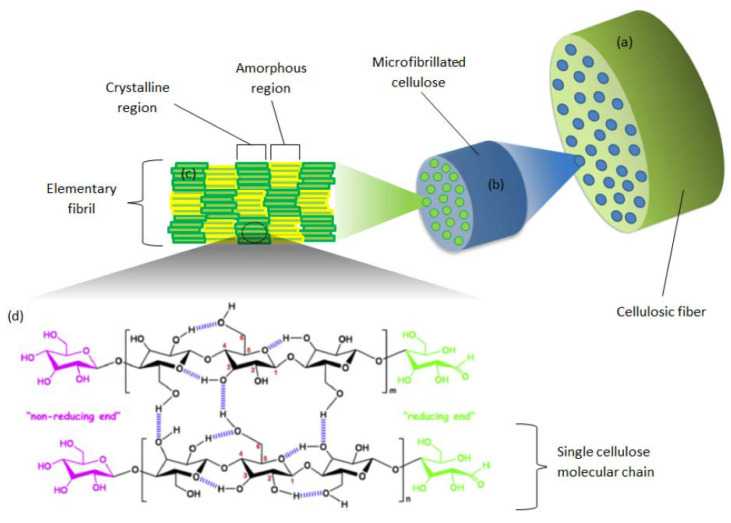
Structural characteristics of cellulosic fibre [75]. Reprinted from International Journal of Biological Macromolecules. Copyright (2019), with permission from Elsevier.

Nanocellulose (NC) is an organic homopolymer, constituted of 1,4-anhydroglucopyranose repeating units (Figure 17). Cellulose nanomaterials are cost-effective, renewable, thermally stable up to 200 °C, and lightweight and provide high strength and stiffness. As a biomaterial of anisotropic shape, good biocompatibility, excellent mechanical properties, and tailorable surface chemistry, it is of high interest for material science and biomedical engineering (wound dressing, nanocarriers for drug delivery, and scaffolds for tissue engineering) [73,74,75,76]. Nanocellulose of defined nano-scale structural dimensions is derived from cellulosic extracts or processed materials. The term covers cellulose nanocrystals (CNCs; also called nanocrystalline cellulose (NCC) or cellulose nanowhiskers (CNWs)), cellulose nanofibrils (CNFs; also known as nano-fibrillated cellulose (NFC)), and bacterial cellulose (BC). They have different crystallinities, surface chemistries, and mechanical properties. Moreover, the rod-like CNCs show concentration-dependent liquid crystalline self-assembly behaviour. Micrometre-long entangled fibrils of CNFs contain both amorphous and crystalline cellulose domains, and their entanglement results in highly viscous aqueous suspensions even at relatively low concentrations (<1 wt%). BC is pure cellulose produced extracellularly by microorganisms.

The development of green nanocomposites based on polylactide and bio-based cellulose nanofillers for different applications, particularly for packaging and biomedical materials, has attracted significant attention [77]. The excellent properties of renewable and bio-based PLA/NC nanocomposites, their cytocompatibility and biodegradability, and their relatively low cost make those materials suitable for biomedical devices with enhanced mechanical performance. The earliest reports can be found in the cited reviews concerning polylactide containing various nanocellulose fillers. The most recent trends, illustrated by the examples given below, include porous and fibrous materials.

Most frequently, the preparation of PLA/nanocellulose composites involves melt processing, wet processing, or their combination. However, the hydrophilic nature of the surface hydroxyl groups on NC particles results in the poor dispersion of nanocellulose in nonpolar media, poor interface adhesion, and agglomeration in the PLA matrix. Thus, the surface modification of reinforcing nanocellulose fillers is often employed to improve their interfacial compatibility with the hydrophobic polylactide matrix. The -OH residues provide abundant active sites for covalent bond formation (oxidation, esterification, etherification, acetylation, carboxymethylation, silylation, and polymer grafting onto the polysaccharide backbone) as well as noncovalent binding. The efficiency of the surface modification strongly depends on the nature of the nanoparticles. Surface treatment may improve both the interfacial adhesion and dispersibility of NC. A significant increase in both mechanical and thermomechanical properties was observed with the addition of surface-treated cellulose. Moreover, hydrophobic nanocellulose can act as a barrier agent. Interfacial bonding and adhesion mechanisms determine the final properties of the polymer composites. Various factors should be taken into account to predict the overall effect. Molecular dynamics simulations characterized the interfacial structure and adhesion behaviour of crystalline nanocellulose in contact with polylactic acid [78]. It was found that adhesion between PLA and the surface of cellulose nanofibers is affected not only by the polarity of the functional groups and hydrogen bonds between CNC and PLA but also the surface roughness. PLA macromolecules can adopt conformations that accommodate the cellulose surface. The rougher the surface of the cellulose planes, the stronger the adhesion. Interestingly, among the four available interfaces, the best adhesion can be achieved at the (010) one due to the greater number of hydrogen bonds formed between the polar (010) surface and the polymer chain. However, the polar (110) and (1−10) surfaces produced similar adhesion work to the nonpolar (100) one (van der Waals effect), which illustrates the amphiphilicity of cellulose.

**Figure 18:**
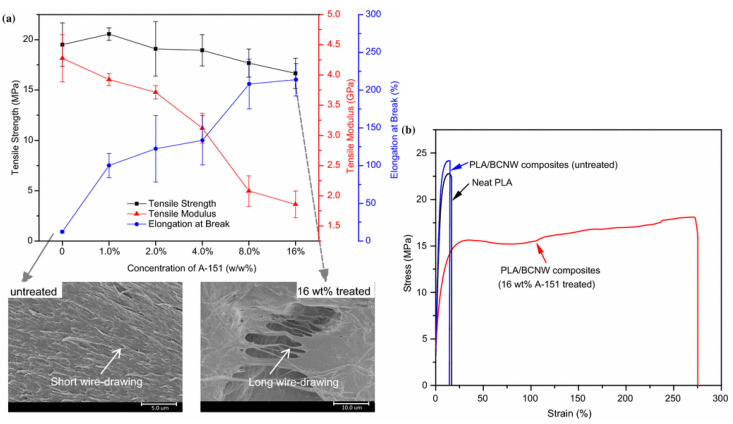
Tensile properties of PLA/composites containing bamboo cellulose nanowhiskers (BCNWs) treated with vinyltriethoxysilane (**a**), and exemplary stress-strain curves (**b**) [80]. Reprinted with permission from Springer Nature: Journal of Materials Science. Copyright (2018).

The functionalization not only enhanced the dispersion of modified NC in polymer composites but also may improve their mechanical properties, supporting specific interactions at the interface between the particles and the matrix. The improved association between the polymer matrix and the filler facilitates stress-transfer and enhances the nanocomposite strength. The amount of cellulose nanofillers determines the crystallinity and mechanical properties of the composite. Well-dispersed nanocellulose fillers act as nucleation sites in PLA, improving the degree of crystallinity and increasing the temperatures of phase transitions. The changes depend on the filler content and its surface chemistry, as well as on the technique used for the preparation of the nanocomposites. For example, ultra-strong PLA/CNC nanocomposites (an ultimate strength of 353 MPa and a toughness of 107 MJ/m^3^) of increased T_g_ (93–95 °C) can be manufactured at a large scale through the surface modification of the nanocrystals, liquid-assisted extrusion, and solid-state drawing [79]. Silylation was applied to improve the surface compatibility between hydrophobic PLA and hydrophilic bamboo cellulose nanowhiskers (BCNWs) [80,81]. The glass transition temperature, crystallinity, tensile strength, and tensile modulus decreased irrespectively of the silane used. However, the maximum elongation at break for the samples containing silanized cellulose increased to 213.8% (16 wt% triethoxyvinylsilane), 111.3% (8 wt% aminopropyltriethoxysilane), 255.3% (8 wt% methacryloxypropyltrimethoxysilane), and 209.8% (8 wt% mercaptopropyltrimethoxysilane) with respect to 12.4% of the untreated PLA/BCNW composite. The results suggest interactions between the ester groups of the PLA backbones and both the functional organic groups and silanol residues of the coupling agents. Many crazes were produced and absorbed most of the tensile energy during mechanical tests (wire-drawing and formation of thin necks) (Figure 18).

It was also shown that the dispersion of the cellulose nanocrystals and formation of a percolation network can be influenced by the molecular weight of the PLA and its crystallizability [76]. Lower CNC percolation concentrations could be obtained in PLA matrices of low molecular weight. CNCs more easily interpenetrate shorter PLA chains dissolved in a good solvent. The CNC percolation concentration could be lowered even more, providing high enantiomeric purity of polylactide chains. Upon solvent evaporation, PLA chains crystallized around the dispersed CNCs, which could prevent the further re-agglomeration of the latter. The rheological properties and thermal stability of such blends were improved.

**Figure 19:**
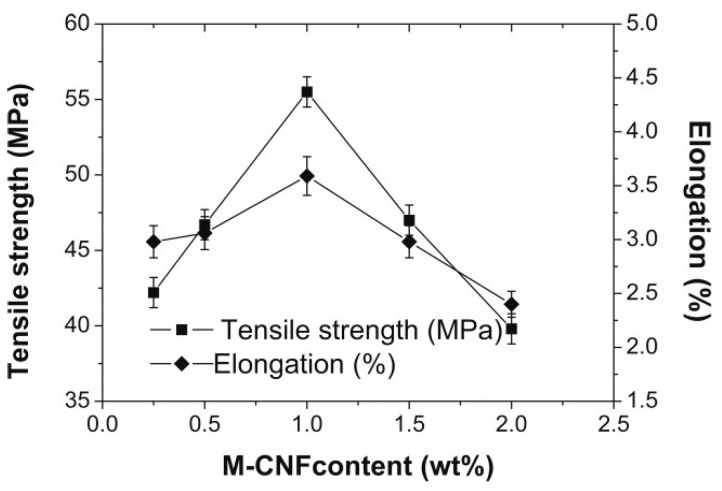
Tensile properties of PLA containing cellulose nanofibrils (CNF) modified with 3-methacryloxypropyltrimethoxysilane [82]. Reproduced with permission of John Wiley & Sons Ltd. Copyright (2012).

3-Methacryloxypropyltrimethoxysilane was also used to modify the surface of cellulose nanofibrils (CNF) that were then used as fillers in poly(lactic acid) [82]. It was found that the silanization slightly decreased the thermal stability of the CNF, but their morphological integrity and rod-like morphology were retained. The treated nanofibrils dispersed well and crossed with each other as a percolated network in the PLA matrix. The tensile strength and elongation of the blends changed with the content of modified CNF. The highest tensile strength was shown for PLA with 1 wt% of the nanocellulose filler (Figure 19). The results were much better than those obtained with BCNW, which confirms the role of percolation for the properties of the composites.

**Figure 20:**
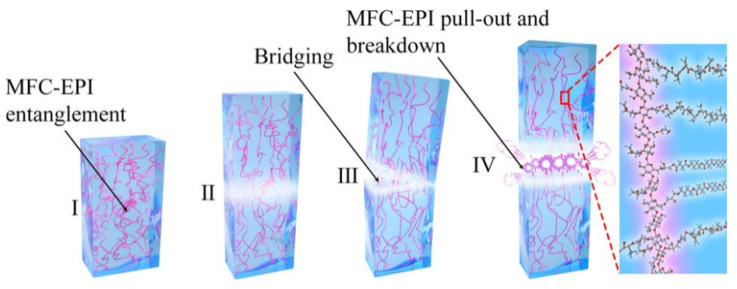
The fracture mechanism explaining the behaviour of PLA/polybutylene succinate (PBS)/epoxidized microfibrillated cellulose (MFC-EPI) composites [83]. Reprinted with permission from ACS Sustainable Chemistry & Engineering. Copyright (2020), American Chemical Society.

A similar concentration dependence was observed when epoxidized microfibrillated cellulose (MFC-EPI) was employed as an interfacial compatibilizer as well as a reinforcement filler in PLA/polybutylene succinate (PBS) blends [83]. The tensile strength and elongation at break of the composite containing 2% MFC-EPI reached 71.4 MPa and 273.6%, respectively. The toughening mechanism was explained by a “bridge” effect of the filler that contributes to energy transfer and dissipation during deformation (Figure 20). The entanglement of long nanofibers of MFC may enhance the load transfer between MFC and the polymer matrix during crack propagation. The interactions were enhanced by chemical cross-linking between the epoxy groups of MFC-EPI and hydroxyl/carboxyl groups of PLA and PBS. MFC-EPI acted as both the compatibilizer and reinforcement filler and endowed the PLA-based materials with high tensile strength and toughness. However, larger amounts of MFC-EPI agglomerated in the polymer matrix, deteriorating the properties of the blend.

The surface of nanocellulose particles can be also rendered with a high content of carboxylic groups through the esterification of the native hydroxyl moieties with citric acid in a solid phase reaction [84]. The modified cellulose nanoparticles were further fibrillated via friction grinding. It strengthened the hydrogen bonding between NC and PLA. The resulting cellulose/PLA composites exhibited a desirable filler-polymer compatibility. The dispersion of the modified cellulose in PLA matrix was good, and the flexural properties of the prepared PLA composites were improved with regard to those of the pristine PLA resin. Maleic anhydride-grafted poly(lactic acid) (PLA-*g*-AMS/MAH) was used as a compatibilizer for the microcrystalline cellulose (MCC)/poly(lactic acid) (PLA) composites [85].

The modification of the nanocellulose filler may also change the gas barrier properties of the PLA nanocomposites [86]. PLA containing well-dispersed lauryl-functionalized cellulose nanocrystals (LNC) exhibited lower gas permeability due to the formation of a rigidified PLA interfacial region with a size comparable to the LNC diameter. The solvent molecules trapped in those regions were released at much higher temperatures than those locked in phase-separated blends.

**Figure 21:**
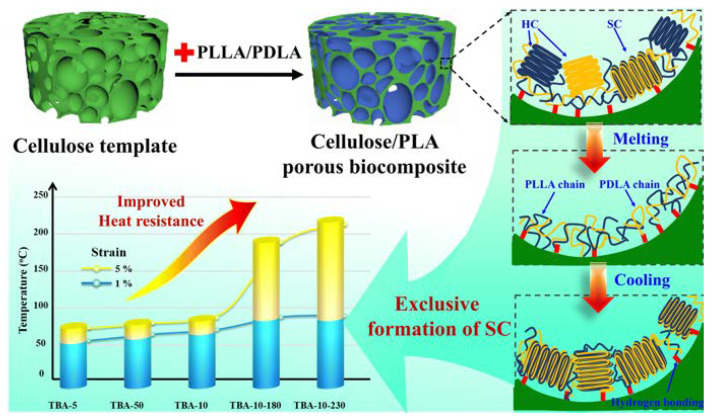
Formation of 3D cellulose/stereocomplex structure (SC) PLA porous biocomposites with improved thermal and mechanical properties [89]. Reprinted with permission from Springer Nature: Cellulose. Copyright (2020).

Biocompatible PLA/nanocellulose composites with good absorbent properties and high mechanical strength can be also used for the preparation of artificial networks and three-dimensional scaffolds with a structure mimicking that of extracellular matrix (ECM). The high porosity of such 3D networks is desirable and beneficial for cell migration as well as nutrient input and metabolite output. Electrospinning technology can be used to create nanofibrous networks, although it is not possible to control their pore size and shape. Well-defined 3D scaffolds made of poly(lactic acid)/regenerated cellulose (PLA/RC) were thus formed using a method that combined freeze-drying and crosslinking [87]. Citric acid was applied as a non-toxic chemical cross-linker to RC nanofibers through esterification with their -OH groups. The resulting bioactive PLA/RC nanofiber-crosslinked scaffolds exhibited a dual pore structure and dimensional stability. Their high water absorption, hierarchical cellular structure, fast recovery from 80% strain, and apatite nucleating capacity indicate good osteogenic potential for bone tissue engineering. Other PLA-based open-pore porous blends suitable for this purpose, with high porosity and interconnectivity as well as superabsorbent ability, were prepared by melt-blending using crosslinked superabsorbent sodium polyacrylate particles (SAP) as a porogen [88]. The SAP particles were leached out from the PLA matrix in an aqueous environment. This generated high and tuneable porosity. The scaffolds allowed the good cell adhesion and proliferation of mouse embryo fibroblasts. It was also shown that similar 3D cellulose/PLA porous bio-composite templates can significantly facilitate PLLA/PDLA stereocomplex crystallization by accelerating the nucleation of SC crystals without the suppression of their growth (Figure 21) [89]. The interfacial hydrogen bonding between cellulose templates and polylactide molecules promoted the formation of precursor racemic helical pairs.

PLA-based blends containing nanocellulose as a reinforcing phase can be also used as an excellent feed material for 3D printing by means of fused deposition moulding (FDM). Unfortunately, the molecular weight of PLA typically becomes reduced after recycling, which limits the reuse of PLA in FDM-based 3D printing. The addition of an epoxy-based chain extender and a reinforcing phase of microcrystalline cellulose (MCC) to regenerated PLA improved its processability and mechanical performance [90]. The addition of 1 wt% nanocellulose enhanced the thermal stability of PLA, increased its crystallization rate, and shortened the crystallization half-time, which is vital for the solidification of a 3D printed object [91]. Polylactic acid/cellulose acetate (PLA/CA) mixtures loaded with antiseptic 1-chloro-2,2,5,5-tetramethyl-4-imidazolidinone were used as biodegradable, low-cost, antibacterial scaffolds prepared by a direct ink writing (DIW) technique [92]. The printability of the DIW inks was improved with the addition of CA due to the formation of a hydrogen-bonded 3D network between PLA and CA.

## 4. PLA Composites with Cyclodextrins

**Scheme 10:**
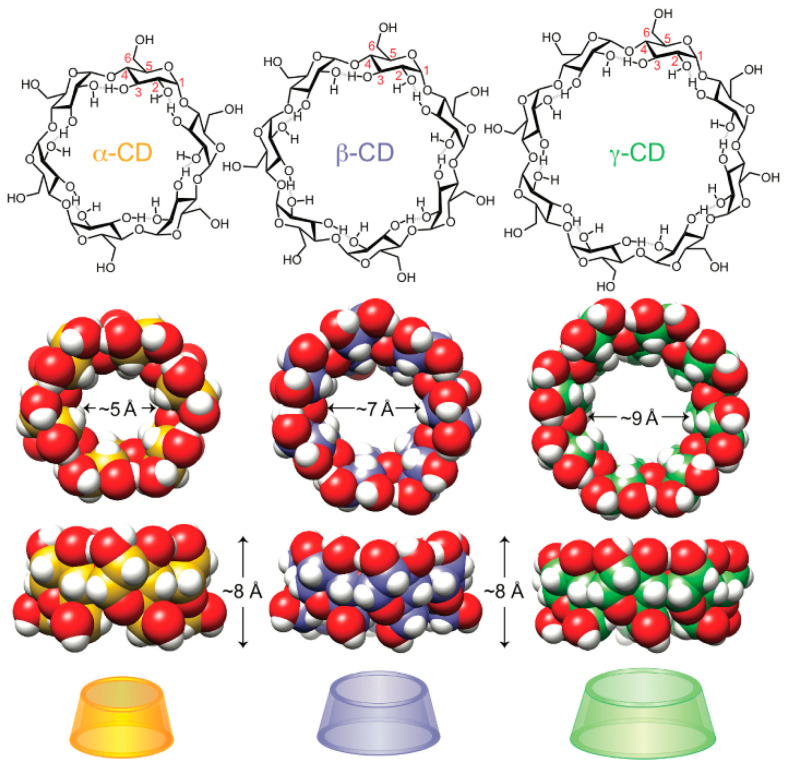
Structural, space-filling, and graphical representations of cyclodextrin molecules: α-CD, β-CD, and γ-CD [96]. Reprinted with permission from Symmetry (MDPI) 2019.

Cyclodextrins (CD) are natural cyclic oligosaccharides produced from starch and consisting of six (*α*-CD), seven (*β*-CD), or eight (*γ*-CD) d(+)-glucose units joined by *α*-1,4-linkages (Scheme 10) [93]. They have a shallow truncated cone shape with a hydrophobic cavity and hydrophilic outer surface. γ-cyclodextrin is the most flexible of the CD molecules, whereas α-cyclodextrin is the most rigid one. The solubility of β-CD in water (18.4 g/L) is low when compared to that of α- and γ-CD (129.5 and 249.2 g/L, respectively) [94]. It appears that intramolecular hydrogen bonding in β-CD results in a tighter crystal structure. Cyclodextrin molecules are amphiphilic, with -CH_2_OH groups linked to the narrower rim and the wider rim displaying -OH groups connected to the glucose rings. The hydrophilic groups are situated on the outside of the molecular cavity whereas the hydrophobic inner surface is lined with ether-like anomeric oxygen atoms and methine units. Thus, cyclodextrin molecules may bind non-polar and suitably-sized aliphatic or aromatic compounds. The binding is driven by the enthalpic and entropic gain related to the reduction in the hydrophobic-aqueous surface and the release of water molecules from the CD cavities. Those specific features make CD effective hosts for a variety of guest molecules (small organic species and macromolecules), resulting in the formation of inclusion complexes (IC) [95,96,97]. The IC formation mechanism involves fitting the guest molecules in the CD cavity through non-covalent interactions (van der Waals interactions and the hydrophobic effect).

Cyclodextrins of all types were used for the formation of “green” IC with polylactide chains. Such polymeric IC may have an important role for constructing supramolecular architectures (molecular tubes or poly(pseudo)rotaxanes). The formed nanometre-scale ordered structures can also be used as nucleating agents to enhance the crystallization of the guest PLA due to the restriction of macromolecular chain motions inside the CD cavities. The introduction of water-soluble cyclodextrins threaded on polymer chains not only affects the crystallization but also modifies the hydrophilicity of polymer surfaces, degradation, and thermal performances. Recently, a significant interest may be also observed in PLA/CD-based materials for the delivery of encapsulated drugs [98,99,100,101,102,103,104] and other medical applications [105,106,107,108].

### 4.1. PLA/α-Cyclodextrins

Threading *α*-cyclodextrin molecules onto PLA chains results in the formation of supramolecular inclusion complexes, organized by non-covalent interactions [109,110,111]. The formation of stable poly(pseudo)rotaxanes was reported when *α*-CD was threaded onto PLLA chains and PLLA-PEG-PLLA triblock copolymers of different molar ratios of [LA]/[PEG] (Tri-1: 23, Tri-2: 31, Tri-3: 45, Tri-4: 54) [110]. For poly(pseudo)rotaxanes of both kinds, threaded onto triblock copolymers or on PLLA, the stoichiometric number of [LA] monomer units and [CD] was found to be 2:1. The complexation strongly reduced the solubility of the polymers.

**Figure 22:**
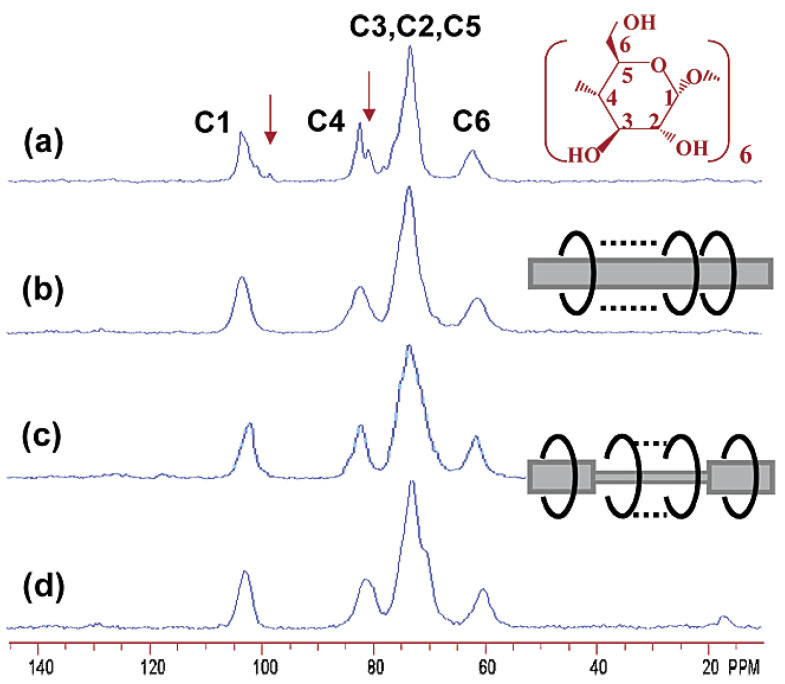
Solid-state ^13^C CP/MAS NMR spectra of R-CD (**a**) and inclusion complexes of PLLA/*α*-CD (**b**), Tri-1/*α*-CD (**c**), and Tri-4/*α*-CD (**d**) [110]. Reprinted with permission from Macromolecules. Copyright (2003), American Chemical Society.

^13^C CP/MAS NMR studies in the solid state indicated that *α*-CD in such polymeric IC adopted more symmetric cyclic conformations, which influenced the splitting of all the C1-6 glucose carbon resonances of the cyclodextrin moieties (Figure 22) [110]. The signals characteristic of the glycoside linkage (98 ppm and 80 ppm) disappeared in the spectra of channel-structured IC. The most symmetric cyclic conformations of *α*-CD were adopted in the inclusion complex with a 2:1 feed ratio. More evidence of IC formation may be obtained by FTIR spectroscopy. A shift of the ν (O-H) mode to higher frequencies can be observed (3389 cm^−1^ for pure *α*-CD and 3420 cm^−1^ for IC) due to the association of OH groups with the guest polymeric chains [110]. The stretching band ν (C-H) (300–2950 cm^−1^) in *α*-CD was also shifted.

**Figure 23:**
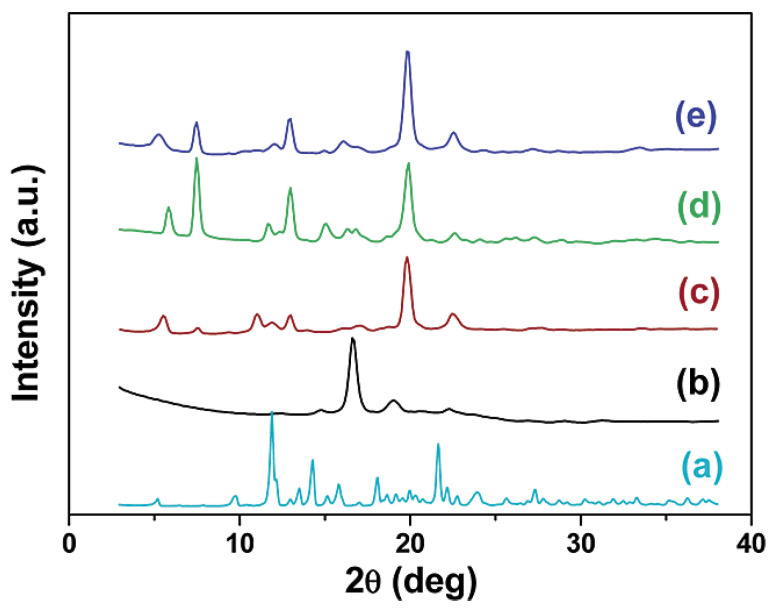
X-ray diffraction patterns for *α*-CD (**a**), PLLA (**b**), poly(ethylene glycol) (PEG)/*α*-CD inclusion complex (**c**), PLLA/*α*-CD inclusion complex (**d**), and Tri-1/*α*-CD inclusion complex (**e**) [110]. Reprinted with permission from Macromolecules. Copyright (2003), American Chemical Society.

The formation of IC with *α*-CD was confirmed by wide-angle X-ray diffraction measurements (exemplary diffractograms are shown in Figure 23). The WAXS patterns of PLA-IC are different from those of PLA and α-CD. A new channel-type crystal structure was formed with the copolymers confined to CD, and the components lost their original crystalline features. The X-ray diffractograms of PLLA/*α*-CD and the Tri-1/*α*-CD inclusion complexes showed patterns different from those of host CD and guest macromolecules [110]. A set of reflections at 2θ = 7.60° (*d* = 11.6 Å), 13.0° (*d* = 6.80 Å), and 20.0° (*d* = 4.44 Å) indicated the formation of a columnar crystalline structure (a hexagonal unit cell with the lateral dimension *a* = 13.6 Å). The strong 210 reflection (2θ = 20.0°) is characteristic of the channel structure of IC crystals containing *α*-CD (the electron density distribution of the core of *α*-CD molecules with a radius of about 5 Å) [110,111].

**Figure 24:**
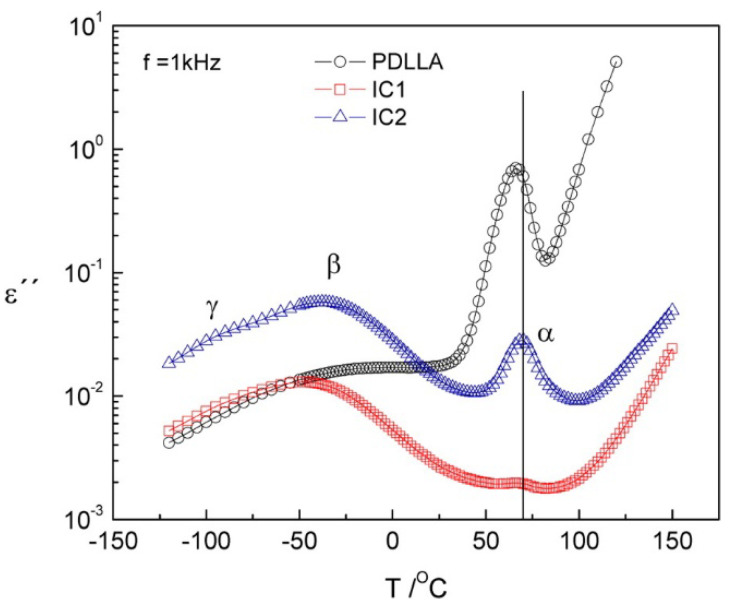
Isochronal (IC) plot of the imaginary part of the complex permittivity at 1 kHz for poly(d,l-lactic acid) (PDLLA) (●), IC1 (■), and IC2 (▲). The ratios of incorporated/initial PDLLA by weight percentage (%, *w*/*w*) were 10/24 (IC1) and 15/46 (IC2) [112]. Reprinted with permission from The Journal of Physical Chemistry B. Copyright (2014), American Chemical Society.

Dielectric relaxation spectroscopy (DRS) studies on poly(D,L-lactic acid) (PDLLA) and its inclusion complexes in α-CD of various ratios of incorporated/initial PDLLA revealed a distinguishably different dynamical response of the PDLLA chains constrained between α-CD from the fraction of macromolecules incorporated inside the channels (Figure 24) [112]. The presence of α-CD molecules depletes the segmental α-process and resolves two sub-T_g_ relaxations. The cooperative motions (α-relaxation) are supressed for PDLLA hosted inside the channels whereas the relaxation of macromolecules situated between α-CD channels is reduced and shifts to higher temperatures (~4.5 °C). A secondary relaxation—the Johari–Goldstein process (βJG-process)—that can be related to the PDLLA confinement effect was also observed in the IC. An additional secondary γ process of length scale inferior to inter- or intra-channel dimensions was detected only in the inclusion complexes.

**Scheme 11:**
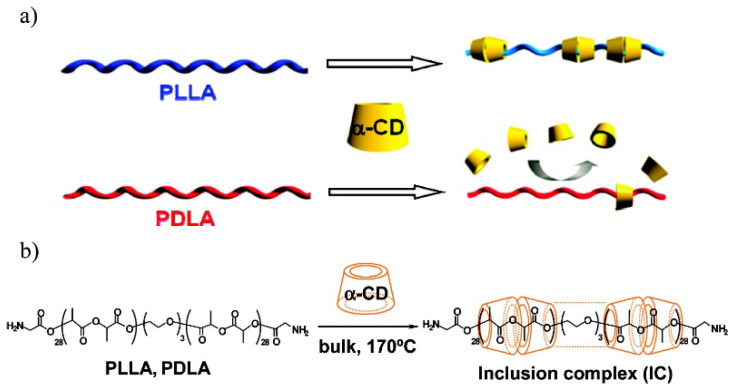
(**a**) Chiral recognition of polylactides by α-CD, and (**b**) preparation of PLA/α-CD inclusion complex [116]. Reprinted with permission from Macromolecules. Copyright (2007), American Chemical Society.

**Figure 25:**
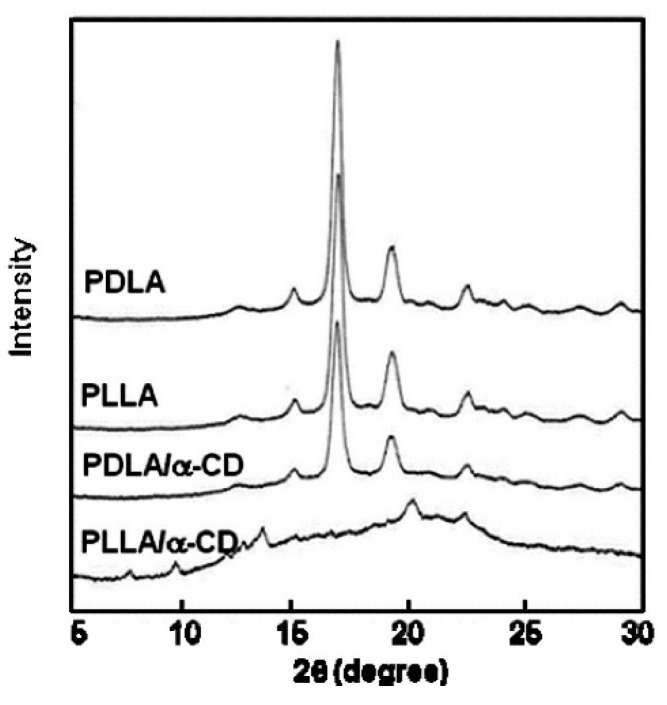
Wide-angle X-ray diffraction patterns of PLLA, PLLA/R-CD poly(D-lactide) (PDLA) and PDLA/R-CD [116]. Reprinted with permission from Macromolecules. Copyright (2007), American Chemical Society.

The optically active CDs are known for their ability to recognize chiral molecules [113,114,115]. Interestingly, Ohya et al. reported that the inclusion complex of α-cyclodextrin with PLLA is preferentially formed compared to that with poly(d-lactide) (PDLA) (Scheme 11) [116]. The recognition of PDLA chains was significant, and they were almost excluded by *α*-CD. The phenomenon was confirmed by tests with (ll)- and (dd)-lactic acid dimer methyl esters. Only (LL) species formed IC with α-CD. The crystalline structures of PLLA/α-CD and PDLA/α-CD were investigated by DSC and X-ray diffraction (Figure 25). The WAXS diagrams of PLLA, PDLA, and PDLA/α-CD featured intensive peaks at 2θ = 17° and 19° (typical for α-crystals of polylactide). PDLA/α-CD showed a melting point around 160 °C (similarly to neat PLLA or PDLA), indicating a crystalline nature of the sample. The diffraction pattern for PLLA/α-CD was characteristic for IC with a columnar structure (2θ = 20°).

Experiments with triblock copolymers—PLLA-PEG-PLLA and PDLA-PEG-PDLA—showed that α-CD molecules were hosting PEG segments irrespectively of the enantiomeric form of the polylactide blocks. It suggests that α-CD can freely slide along the PDLA segments and that the fitting between the polymer width and diameter of the CD cavity is not the only factor that governs the IC formation. It was therefore concluded that the reason for the chiral recognition is not the polymer chain steric hindrance (similar for PLLA and PDLA) but the thermodynamic stability of the diastereomeric IC. Chiral fitting with the α-CD cavity is more favourable to PLLA and allows the linking of several cyclodextrine molecules through hydrogen bonds. The molecules of α-CD have to rotate unidirectionally on sliding down the helical chain of PLLA. This phenomenon may be exploited for the design of molecular motors.

**Figure 26:**
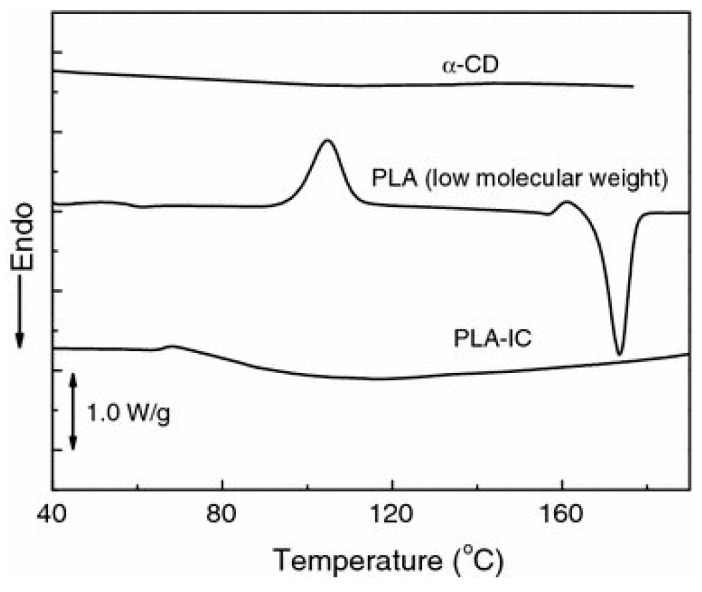
DSC traces of *α*-CD, PLA, and PLA-IC during heating at 10 °C/min [111]. Reprinted with permission from Springer Nature: Polymer Bulletin. Copyright (2013).

The *α*-CD host polylactide guest IC with a channel-type structure had different thermal characteristics to the parent polylactide [109,110,111]. No cold crystallization and melting can be detected if the PLA chains are included inside the *α*-CD voids (Figure 26). Small amounts of IC may exert a nucleation effect and promote the crystallization of the PLA matrix during both the non-isothermal and isothermal crystallization experiments [111]. Upon cooling from melt (5 °C/min), an exotherm was observed at about 94 °C, suggesting that IC particles can accelerate the nucleation. If the PLA contained free α-CD (not IC) then the nucleation effect was small. It was also noted that *α*-CD may act as a nucleating agent for the crystallization of PLLA from the solution with the formation of spherulitic structures [117]. The size of the spherulites in the cast film depended on the amount of *α*-CD and the casting rate. Very small crystallites were formed in the presence of IC and on fast casting. 

### 4.2. PLA/β-Cyclodextrins

The feeding ratio between β-CD and PLA, as well as the molecular weight of the latter, has a significant effect on the formation of IC prepared by the solution ultrasonic technique [118]. IC with PLA of Mn ≥ 500 kg/mol agglomerated in water, but if the Mn was <100 kg/mol then the IC was soluble in H_2_O. Both IC had lower T_g_ and were more hydrophilic than those of pure PLA. FTIR spectra showed a clear difference between the IC and the components *β*-CD and PLA. The ν (O-H) stretching modes of *β*-CD shifted to higher frequencies when the inclusion complexes were formed, indicating physical interactions between the components. The ν (C=O) stretching band of PLA (1750 cm^−1^) shifted to 1757 cm^−1^ and weakened in the spectrum of IC. Wide angle X-ray diffraction patterns revealed a specific crystal structure of IC that differed from those characteristic of neat *β*-CD and PLA. The T_g_ of the inclusion complex was lower than that of pure PLA, and the decrease was proportional to the amount of *β*-CD. The drop in the glass transition temperature was explained in terms of IC supramolecular structure formation with the molecular rings of *β*-CD hindering interactions between the PLA chains. The thermal stability of the IC was slightly lower than that of pure PLA.

The hydrogen bonds between polylactide and *β*-CD were confirmed by a shift of the ν (O-H) stretching IR mode of *β*-CD (3386 cm^−1^) towards a lower wavelength in the IC spectrum (3304 cm^−1^) [119]. Additionally, a shift in the ν (C-H) band of *β*-CD was noted. The association behaviour can be explained by the presence of an ordered phase with different chain conformation, as indicated by the ν (C=O) stretching band of PLLA. The second derivative of this band changes markedly in IC, with a decrease in the trans-gauche conformation component. Those changes may be related to the formation of hydrogen bonding between the blended molecules.

The absence of the hydrogen bonding hinders the propensity towards IC formation in polylactide/cyclodextrin systems. The addition of methyl-β-cyclodextrin (Me-β-CD) lowered the crystallinity of the PLLA and enhanced the mobility of the polyester chains [120]. The addition of Me-β-CD increased the amorphous component of the PLLA. The plasticizing of PLLA by Me-β-CD resulted in an improvement of the drawability of the composite (1000% at 60 °C in the presence of 17% of Me-β-CD). In situ measurements using combined differential scanning calorimetry (DSC) and Raman spectroscopy revealed a lowering of the glass transition temperature, cold crystallization temperature, and melting point. As no IC was formed and the amount of the amorphous phase increased, the thermal stability of the PLLA/Me-β-CD blend was lower than that of neat PLLA.

The mechanism of polylactide/β-cyclodextrin inclusion complex formation and the effects of its incorporation in the PLA matrix were investigated [121]. The changes in the thermal stability, surface morphology, and barrier and mechanical properties were studied for the composites at varying IC and β-CD concentrations. Samples admixed with neat β-CD featured agglomerates of the β-cyclodextrin molecules while PLA/IC composites had uniform structures. The reason was poor interfacial interactions between the polyester macromolecules and β-CD moieties. The structural variations were reflected in different thermal stabilities. The T_g_ and T_c_ of the studied PLA composites were shifted to higher temperatures upon increasing the concentration of IC or β-CD. The composite films containing larger amounts of IC or β-CD exhibited higher oxygen and water vapour permeability but were less flexible and had lower tensile strength than neat PLA.

High molecular weight polymer chains of stronger steric hindrance cannot easily penetrate the cavities of cyclodextrins; thus, the macromolecules tend to form partial inclusion complexes with small stoichiometric ratios. Nevertheless, the partial inclusion complexes formed between β-CD and high molecular weight PLLA crystallized better than neat PLLA. An improved mechanical performance and thermal stability were also demonstrated [122].

### 4.3. PLA/γ-Cyclodextrins

A poly(lactic acid) and γ-cyclodextrin inclusion complex was obtained by ultrasonic co-precipitation and used as a precursor for the preparation of its benzyl-hydrazide derivative that was subsequently applied as a nucleating agent for PLA composites [123]. PLA chains penetrated into the cavity of the γ-CD cone, which resulted in a tunnel-shaped crystalline structure. For PLA/γ-CD IC, two prominent X-ray diffraction peaks were formed (2θ = 7.5° and 19.6°). The latter indicated the channel-like structure characteristic of crystalline necklace-like IC with a fingerprint diffraction feature at 2θ = 7.5°. The comparative FTIR analysis of PLA-IC and neat PLA and γ-CD showed that the interactions between those two components were strong. The peak corresponding to symmetric and antisymmetric O-H stretching modes of γ-CD (3405 cm^−1^) was shifted to 3415 cm^−1^ in PLA-IC. This confirmed a non-covalent nature of the interactions between the γ-CD and PLA backbones in the inclusion complex. Moreover, the carbonyl stretching (*ν*, C=O) appeared at 1760 cm^−1^ in the neat PLA and at 1768 cm^−1^ in the IC (noticeably weakened). Calorimetric studies of PLA/PLA-IC showed that T_c_ and T_m_ trended towards lower-temperature regions. It suggested that PLA-IC could form hydrogen bonds with PLA and break the intermolecular interactions in PLA. PLA/γ-CD IC was also modified via surface-initiated atom transfer radical polymerization to obtain poly(lactic acid)-*γ*-cyclodextrin inclusion complex-poly(glycidyl methacrylate) (PLA-IC-PGMA) [124].

## 5. Conclusions

Polylactide nanocomposites, composites, and blends containing specific organic compounds (arylamides and arylhydrazides, 1,3:2,4-dibenzylidene-d-sorbitol, or orotic acid), humic and fulvic acids, nanocellulose, and cyclodextrins have been reviewed. Supramolecular interactions operating in those blends play a very important role in the properties of those novel hybrid materials. They differ from other PLA-based hybrids containing inorganic additives or composites of PLA and graphene or carbon nanotubes.

Aryl nucleators containing amide or hydrazide linkages are capable of crystallization in the polylactide melt. The formation of nanofibrils/nano-objects upon the self-organization of these moieties exploits two recognition motifs: hydrogen bonds and π-π interactions between the molecules taking part in the formation of supramolecular structures and intermolecular hydrogen bonds linking those formations and the polyester backbones. Macromolecular nucleators (humic/fulvic acids and nanocellulose) not only help in the formation of crystal nuclei but also improve the mechanical properties of the PLA. Host–guest effects in blends containing inclusion complexes of PLA and (α/β/γ)-cyclodextrins change the mobility of the polyester chains. The formed structures may also enhance the polylactide crystal growth, but they have much larger potential. Treading cyclodextrins onto PLLA backbones may be used for the design of biocompatible molecular rotors. Interestingly, chiral recognition was observed for the PLLA/PDLA mixtures and α-CD.

The performance of PLA can be improved because of those specific interactions that modify the organization of the polymer matrix. The “soft templating” may change the conformation of segments in PLA chains and influence the nucleation of polymer crystals, thus enormously enhancing the crystallization process. Despite the templating, the type of crystal structure is not changed, except that the well-organized α-crystals are more easily formed in such systems. The influence of supramolecular nucleators is of exceptional importance, as the crystallinity of PLA plays a significant role in its mechanical and barrier properties, biodegradability, and thermal stability. Many supramolecular systems described in this review are biocompatible and can be used for biomedical purposes.
